# Enhanced Environmental PFAS Characterization Using a Virtual High-Resolution Mass Spectral Library Generated by Transfer Learning-Based Neural Network

**DOI:** 10.1021/acs.est.6c05733

**Published:** 2026-06-10

**Authors:** Yi-Chi Chen, Hsin-Yi Wu, Man-Ni Zhuang, Chen-Ming Yi, Wei-Sheng Wu, Pao-Chi Liao

**Affiliations:** † Department of Environmental and Occupational Health, College of Medicine, 549736 National Cheng Kung University, Tainan 704, Taiwan; ‡ Instrumentation Center, 34912 National Taiwan University, Taipei 106, Taiwan; § Institute of Computer and Communication Engineering, National Cheng Kung University, Tainan 701, Taiwan; ∥ Department of Electrical Engineering, National Cheng Kung University, Tainan 701, Taiwan

**Keywords:** PFAS, high-resolution mass spectrometry, mass spectral library, artificial neural network, transfer learning

## Abstract

Per- and polyfluoroalkyl substances (PFAS) represent a critical class of persistent environmental contaminants with significant ecological and human health implications. However, the rapid emergence of novel PFAS has far outpaced the development of reference mass spectral databases. Here, Neural Per- and Polyfluoroalkyl Substances Mass Spectrometry (NPFAS-MS), a transfer learning-based neural network model, was developed to predict PFAS-specific high-resolution mass spectra. NPFAS-MS was fine-tuned from a pretrained model using PFAS tandem mass (MS/MS) spectra. NPFAS-MS outperformed other *in silico* spectral prediction models for PFAS spectra prediction across multiple spectral similarity metrics. In library searching tasks, libraries generated by other spectral prediction models showed top-1 recall between 42.1% and 55.4%, while NPFAS-MS demonstrated 71.1%. Applying the virtual PFAS mass spectral library generated with NPFAS-MS using 10,553 PFAS structures from the U.S. EPA and NORMAN databases to groundwater and aqueous film-forming foam (AFFF) samples revealed more potential PFAS than other mass spectral databases. Specifically, 38 potential PFAS were annotated in AFFF products and 40 in groundwater samples. NPFAS-MS enabled characterization of emerging PFAS, including ultrashort-chain, unsaturated, and substituted derivatives in environmental matrices. This advancement enables comprehensive environmental monitoring of rapidly evolving PFAS contamination. NPFAS-MS and associated resources were deployed as a web-based tool at https://cosbi10.ee.ncku.edu.tw/NPFAS_MS/, enabling both structure-to-spectrum prediction and library searching against 31,659 predicted PFAS spectra.

## Graphical abstract

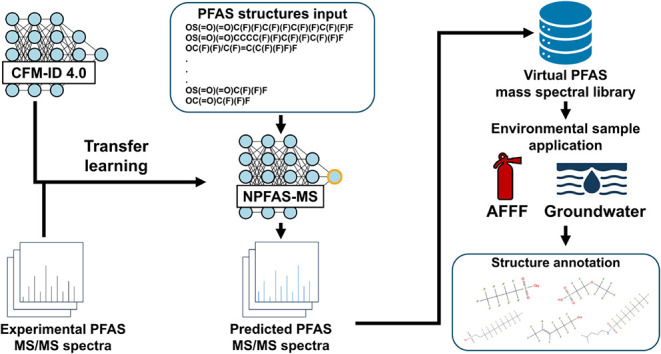

## Introduction

Per- and polyfluoroalkyl substances (PFAS) are a group of anthropogenic compounds characterized by their exceptional stability, persistence, and surface-active properties. Due to these unique characteristics, PFAS have been extensively utilized in industrial and consumer applications, including protective coatings, food packaging materials, and aqueous film-forming foams (AFFF).^1–4^ The widespread use of PFAS has led to their ubiquitous detection in various environmental matrices, including soil, surface and groundwater, and atmospheric systems.^5–7^ The carbon–fluorine bonds inherent in PFAS structures contribute to their extraordinary environmental persistence and bioaccumulation potential.^8,9^ Beyond their persistence, PFAS exposure is associated with significant toxicological risks and adverse health effects in both aquatic organisms and humans.^10–14^

However, as regulatory restrictions have been implemented for well-studied long-chain PFAS such as perfluorooctanoic acid (PFOA) and perfluorooctanesulfonic acid (PFOS) under the Stockholm Convention, the chemical industry has increasingly shifted toward alternative PFAS formulations, including ultrashort-chain (C < 4) variants and structurally diverse derivatives.^15^ Emerging PFAS alternatives have been detected across multiple environmental matrices, including chloro-perfluoroalkanesulfonates and unsaturated perfluoroalkanesulfonates in AFFF-contaminated groundwater,^16^ and novel perfluorobutane sulfonamido derivatives in semiconductor manufacturing effluents.^17^ Owing to its high sensitivity and selectivity, liquid chromatography–high-resolution mass spectrometry (LC-HRMS) has been widely applied to the analysis of environmental contaminants and exposome-related chemicals.^18,19^ Reference mass spectral databases searching serves as one of the primary structural characterization approaches in current LC-HRMS-based analysis, enabling the large-scale and rapid annotation of potential compounds present in samples. This approach typically involves comparing experimental MS/MS spectra against reference spectra from known compounds in the mass spectral databases.^20^ Unfortunately, the rapid emergence of novel PFAS has far outpaced the development of comprehensive reference mass spectral databases. The magnitude of this analytical challenge becomes apparent when considering the limitations of reference databases. For example, mzCloud,^21^ a major reference mass spectral database, contains MS/MS spectra for fewer than 200 PFAS structures, whereas the U.S. EPA CompTox Chemicals Dashboard lists over 10,000 different PFAS structures.^22^

To address these analytical limitations, recent advances in machine learning and deep learning have demonstrated significant potential for overcoming these analytical challenges through computational approaches for predicting MS/MS spectra.^23^ Compound-to-mass spectrum (C2MS) prediction tools predict MS/MS spectra from chemical structures. These approaches learn structure-spectrum relationships directly from experimental data, enabling spectral prediction for compounds without requiring predefined fragmentation patterns.^24^ NEIMS employs a bidirectional neural network architecture for electron ionization mass spectrometry prediction with rapid computational speed.^25^ CFM-ID utilizes competitive fragmentation modeling based on a Markov stochastic process to predict MS/MS spectra.^24^ Graph neural networks are implemented in FIORA to leverage local molecular neighborhoods around chemical bonds for fragment ion prediction.^26^ These C2MS approaches have been applied to various compound classes, including PFAS analysis. Getzinger et al. applied CFM-ID to create a virtual MS/MS spectral library by combining PFAS structures from EPA and in-house databases with their predicted biotransformation products,^27^ demonstrating the potential of *in silico* prediction tools for expanding PFAS identification capabilities. Although these C2MS approaches have shown promising results, their performance can be significantly enhanced through the application of transfer learning strategies. Transfer learning enables the adaptation of generic models to specific chemical domains through fine-tuning with target-domain data. This approach significantly enhances model performance on specific tasks by transferring knowledge from large general data sets, enabling the development of highly specialized predictive functions with greater accuracy for specific compound classes.^28^ Recent successful applications demonstrate the effectiveness of this approach, with NPS-MS demonstrated robust performance for new psychoactive substances characterization and DeepCDM showing enhanced accuracy for chemically derived molecules, both through transfer learning from generic mass spectral prediction models.^29,30^ However, MS/MS spectral libraries specifically tailored for PFAS remain limited, creating considerable challenges for reliable characterization of this important class of environmental contaminants.

The extensive environmental distribution of PFAS and their potential health impacts necessitate enhanced analytical capabilities for comprehensive environmental monitoring. In response to this critical need, Neural Per- and Polyfluoroalkyl Substances Mass Spectrometry (NPFAS-MS) was developed as a transfer learning-based neural network model designed to expand PFAS annotation capabilities in environmental systems. While existing generic prediction tools such as CFM-ID and FIORA have shown promise, the unique structural characteristics and fragmentation patterns of fluorinated molecules present specific challenges for accurate environmental detection. NPFAS-MS employs transfer learning to fine-tune the general MS/MS spectral prediction model CFM-ID 4.0 using a curated set of PFAS MS/MS spectra. This creates an optimized model for PFAS MS/MS spectra prediction that overcomes limitations of reference mass spectral databases and expands annotation capabilities for emerging and previously unannotated PFAS. An extensive virtual mass spectral library was constructed, containing 31,659 predicted MS/MS spectra for 10,553 PFAS structures from the U.S. EPA and NORMAN databases. This library was applied to characterize PFAS in environmental samples from AFFF commercial products and groundwater. To facilitate broader application, we developed a user-friendly web-based tool that supports both PFAS MS/MS spectral prediction and library searching for PFAS annotation. The enhanced characterization capabilities demonstrated by this approach offer significant potential for improving environmental PFAS monitoring.

## Materials and Methods

### Chemicals and Reagents

The 51 authentic reference PFAS standards (detailed in the Supporting Information (SI), Table S1) were purchased from Wellington Laboratories, Inc. (Guelph, Ontario, Canada), Chiron AS (Trondheim, Norway), AK Scientific, Inc. (Union City, CA, USA), Toronto Research Chemicals (North York, Ontario, Canada) and Dr. Ehrenstorfer GmbH (Augsburg, Germany). Methanol (MeOH, purity ≥99.9%) was purchased from Merck (Darmstadt, Germany). Acetonitrile (ACN, purity ≥99.9%) and ammonium hydroxide (30% in water) were purchased from J.T. Baker (Phillipsburg, NJ, USA). Formic acid (purity ≥98%) and ammonium acetate (28.0–30.0%) were purchased from Sigma-Aldrich (St. Louis, MO, USA). Solid-phase extraction (SPE) cartridges (WAX, 150 mg, 6 cc) were purchased from Waters Corporation (USA).

### Environmental Sample Collection and Preparation

For AFFF samples analysis, eight samples were obtained from four different suppliers, all consisting of 3% AFFF concentrates as detailed in Table S2. These commercial concentrates are typically diluted with water in a 97:3 ratio (water to concentrate) for firefighting applications.^31^ Groundwater samples were collected as grab samples in June 2025 from two different locations in northern Taiwan. The samples were stored in 2 L high-density polyethylene (HDPE) bottles, then transported to the laboratory and stored at 4 °C until sample preparation and analysis.

Each AFFF sample was thoroughly mixed by hand shaking until substantial foaming occurred and stratification became static. The homogenized samples were then transferred into polypropylene (PP) centrifuge tubes. For analysis, AFFF samples were diluted with MeOH at a ratio of 1:19 (v/v) to reduce matrix effects and ensure compatibility with the analytical system. The diluted samples were centrifuged at 20,000 *g* for 15 min at 4 °C to remove particulates and separate phases. Following centrifugation, 60 μL of the supernatant was collected for LC-HRMS analysis.

The groundwater sample preparation procedure was performed using SPE based on established EPA Method 1633A protocols with modifications.^32^ Briefly, 60 mL of pooled groundwater was manually extracted and purified using Oasis WAX cartridges (6 cc, 200 mg). Before sample loading, cartridges were conditioned and equilibrated sequentially with 15 mL of 0.1% methanolic ammonium hydroxide followed by 5 mL of 0.3 M formic acid in deionized water (DIW). The pooled groundwater sample (60 mL, pH adjusted to 6–7) was then loaded into the preconditioned cartridges. After sample loading, cartridges were washed twice with 5 mL of 0.05 M formic acid in DIW to remove interfering substances. Targeted analytes were eluted using 5 mL of MeOH followed by 5 mL of 0.1% methanolic ammonium hydroxide. The combined eluates were evaporated under a stream of nitrogen to 100 μL and reconstituted to 200 μL with MeOH. The final extracts were vortexed, centrifuged at 20,000 *g* for 15 min at 4 °C, and the supernatant was collected for LC-HRMS analysis, resulting in a concentration factor of 1000.

### LC-HRMS Nontargeted Analysis

LC–HRMS analysis was performed using a Dionex UltiMate 3000 UHPLC coupled with a Q Exactive Plus mass spectrometer (Thermo Fisher Scientific, Bremen, Germany) operating in negative electrospray ionization (ESI) mode. Target compounds were separated using a Phenomenex Luna Omega polar C18 column (100 mm × 2.1 mm, 1.6 μm, Torrance, CA, USA) maintained at 40 °C. The mobile phase consisted of two eluents: (A) 2 mM ammonium acetate in 95:5 water/acetonitrile and (B) acetonitrile. A 5 μL sample injection was used for all analyses. For the AFFF samples, a gradient was used at constant 0.35 mL/min flow rate: 2% B for 0–1 min; 2–95% B in 1–12 min; 95% B in 12–14 min; 95–2% B in 14–15 min, finally maintain 2% B for 1 min (15–16 min) for column re-equilibration. For the groundwater samples, a gradient was used at constant 0.35 mL/min flow rate: 2% B for 0–1 min; 2–99% B in 1–11 min; 99% B in 11–13 min; 99–2% B in 13–13.01 min, finally maintain 2% B in 13.01–14 min for column re-equilibration.

For the HRMS analysis, full scan MS data were acquired from *m*/*z* 80 to 1,000 at a resolution of 70,000, with an automatic gain control (AGC) of 3 × 10^6^ and a maximum dwell time of 200 ms. Following feature prioritization, samples were reinjected to acquire MS/MS spectra. MS/MS spectra were acquired in data-dependent acquisition (DDA) mode using high-energy collisional dissociation (HCD) fragmentation with normalized collision energy (NCE) of 25 and 40, an isolation window of 1.6 Da, an AGC of 1 × 10^6^, a maximum dwell time of 50 ms, and a resolution of 15,000.

For the feature prioritization in nontargeted analysis, full scan MS data from AFFF and groundwater samples were individually processed using MS-DIAL (version 5.1)^33^ for peak alignment. For each sample type, detected features were filtered against the 10,553 PFAS structures of interest from the U.S. EPA and NORMAN databases, as well as Kendrick mass defect (KMD) filtering to detect potential PFAS-related features based on their characteristic CF_2_ repeat units. The CF_2_-normalized KMD for all features was calculated using eqs 1 and 2:^34^

1 $$\mathrm{Kendrick mass}\;\left(\mathrm{KM}\right)=\mathrm{measured mass}\times \frac{\mathrm{nominal mass of repeating unit}}{\mathrm{exact mass of repeating unit}}$$

2 KMD=nominal mass−KM

Features meeting either criterion were prioritized for MS/MS acquisition. To eliminate potential contamination and background interference, features detected in blank samples were excluded from further analysis. The remaining features were subjected to MS/MS analysis and searched against a virtual PFAS mass spectral library. A detailed workflow was provided in Figure S1.

### NPFAS-MS Model Development and MS/MS Spectra Data Sets

NPFAS-MS adopted the model architecture of CFM-ID.^24^ CFM-ID can precalculate the theoretically possible fragments from each structure and compute the probability of each fragment. Each parameter is optimized by learning from known structures and their corresponding MS/MS spectra, ultimately enabling the prediction of MS/MS spectra for input molecular structures. CFM-ID can predict MS/MS spectra at three different collision energy (CE) levels (low, medium, and high) to correspond with the spectra generated using different CE in actual sample analysis. In this study, a transfer learning strategy was applied to CFM-ID 4.0 [M – H]^−^, a generic pretrained model trained on a large data set of MS/MS spectra. By fine-tuning the pretrained model with PFAS MS/MS spectra, NPFAS-MS was enabled to accurately predict PFAS MS/MS spectra. The configuration parameters of the pretrained CFM-ID 4.0 model and the fine-tuned NPFAS-MS model are summarized in Tables S3 and S4, respectively.

For the transfer learning and model evaluation, a data set containing 415 MS/MS spectra corresponding to 140 PFAS structures was first divided into six different categories based on structural features. Each category was then randomly split into a training set and a hold-out test set, with an approximate 70:30 ratio. PFAS MS/MS spectra from two sources were utilized. First, 153 MS/MS spectra were acquired by analyzing 51 PFAS reference standards using a Q Exactive Plus mass spectrometer at three different NCE (20, 35, and 50).^35^ Second, 1,752 MS/MS spectra were additionally collected from MassBank of North America (MoNA),^36^ National Institute of Standards and Technology (NIST) DIMSpec database,^37^ and MassBank,^38^ covering 89 additional PFAS structures (not included in the reference standards above) analyzed under various CE using negative ESI mode with either quadrupole time-of-flight (Q-TOF) or Orbitrap mass spectrometers.

To address the issue of imbalanced data sets arising from online public database collections, the MS-Clustering strategy was implemented to select representative spectra.^39^ To standardize energy units, spectra with NCE values were converted using the calculation method described in Proteomics News (https://proteomicsnews.blogspot.com/2014/06/normalized-collision-energy-calculation.html). For each PFAS structure, MS/MS spectra were ranked by converted CE values and divided into three equal groups (low, medium, and high CE), and the MS-clustering strategy was performed within each group. Within each cluster, the spectrum exhibiting the highest similarity to other spectra in the same cluster was selected as the representative spectrum. Detailed results of MS-clustering were shown in Figure S2. Finally, the MS-Clustering-processed data set was combined with the in-house data set from reference standard analysis, resulting in a final data set of 415 MS/MS spectra covering three different CE levels (low, medium, and high) corresponding to 140 PFAS structures for NPFAS-MS model training and evaluation. All spectra were preprocessed to remove peaks with relative abundance below 3% to ensure spectral quality.

### Model Evaluation and Statistical Analysis

The predictive performance of NPFAS-MS for PFAS MS/MS spectra prediction was assessed using a hold-out test set comprising 121 spectra from 42 PFAS structures. To evaluate the similarity between predicted and experimental spectra, four complementary spectral similarity metrics were employed: dot product (also known as cosine similarity), entropy similarity, dice coefficient, and precision.

Dot product was computed according to (eq 3),^38^ offering a normalized measure of spectral similarity that considers the angular relationship between predicted and experimental intensity vectors.

3 $$\mathrm{Dot product}\left(I_{q},I_{l}\right)=\frac{\overset{M_{\max}}{\underset{k=1}{\sum }}m_{k}^{2}I_{\mathrm{qk}}^{0.5}\cdot m_{k}^{2}I_{lk}^{0.5}}{\sqrt{\overset{M_{q}}{\underset{k=1}{\sum }}{\left(m_{k}^{2}I_{qk}^{0.5}\right)}^{2}}\cdot \sqrt{\overset{M_{l}}{\underset{k=1}{\sum }}{\left(m_{k}^{2}I_{lk}^{0.5}\right)}^{2}}}$$

Entropy Similarity was calculated according to (eq 4).^40^ It assesses the change in entropy that occurs when mixing the predicted and experimental spectra into a single combined spectrum.

4 $$\mathrm{Entropy similarity}=1-\frac{2S_{\mathrm{AB}}-S_{A}-S_{B}}{\ln4}$$

Dice Coefficient was determined using (eq 5),^24^ which focuses on the overlap of detected fragment ions between predicted and experimental spectra. This binary-based metric assesses the model’s ability to accurately predict the presence or absence of a specific *m*/*z*, regardless of intensity accuracy.

5 $$\mathrm{Dice coefficient}\left(I_{q},I_{l}\right)=\frac{2\overset{M_{\max}}{\underset{k=1}{\sum }}\left(I_{qk}>0\cap I_{lk}>0\right)}{\overset{M_{\max}}{\underset{k=1}{\sum }}\left(I_{qk}>0\right)+\overset{M_{\max}}{\underset{k=1}{\sum }}\left(I_{lk}>0\right)}$$

Precision was calculated according to (eq 6),^24^ measuring the percentage of correctly predicted peaks:

6 $$\mathrm{Precision}\left(I_{q},I_{l}\right)=\frac{\overset{M_{\max}}{\underset{k=1}{\sum }}\left(I_{qk}>0\cap I_{lk}>0\right)}{\overset{M_{\max}}{\underset{k=1}{\sum }}\left(I_{Ik}>0\right)}$$

In these calculations, *m*_*k*_ represents the *m*/*z* of fragment ions, while *I*_q*k*_ and *I*_l*k*_ denote the corresponding intensities in the predicted and experimental spectra, respectively. The parameters *M*_q_ and *M*_l_ indicate the highest nonzero indices in each spectrum, with *M*_max_ representing the maximum of these two values. For the entropy similarity calculation, *S*_AB_ represents the spectral entropy of the mixed spectrum (1:1 combination of spectra A and B), while *S*_A_ and *S*_B_ denote the spectral entropies of the individual predicted and experimental spectra, respectively.

Statistical significance testing was performed to compare the performance of four spectral prediction models (NPFAS-MS, CFM-ID 4.0, FIORA, and PFAScreeneR) across the four spectral similarity metrics. One-way analysis of variance (ANOVA) was conducted to evaluate whether significant differences existed among the models for each metric. For metrics showing significant differences in one-way ANOVA (*p* < 0.05), Dunnett’s test was employed to compare each of the three existing methods (CFM-ID 4.0, FIORA, and PFAScreeneR) against NPFAS-MS as the reference group. To evaluate model stability across five repeated random splits, one-way ANOVA was also used to assess whether significant differences existed among the five splits for each spectral similarity metric. All statistical analyses were performed using R statistical software (version 4.4.1).

## Results and Discussion

### Overview of NPFAS-MS Development and Application

The rapidly emerging PFAS in the environment have already exceeded the PFAS MS/MS spectra available in existing reference mass spectral databases, as indicated by the number of PFAS listed in the U.S. EPA and NORMAN databases. This makes a comprehensive analysis of PFAS particularly challenging. In this study, NPFAS-MS was developed, a model capable of predicting high-resolution MS/MS spectra of PFAS. The development and application of NPFAS-MS comprise three components: First, NPFAS-MS, a specialized neural network model for PFAS MS/MS spectrum prediction, was developed through a transfer learning approach by fine-tuning a pretrained negative ESI model (CFM-ID 4.0) with PFAS experimental MS/MS spectra (Figure 1A). Second, a virtual PFAS spectral library containing 31,659 predicted MS/MS spectra for 10,553 PFAS structures combined from the U.S. EPA and NORMAN databases was generated by NPFAS-MS (Figure 1B). Third, MS/MS spectra acquired from groundwater and AFFF samples were searched against the virtual library (Figure 1C).

**Figure 1:**
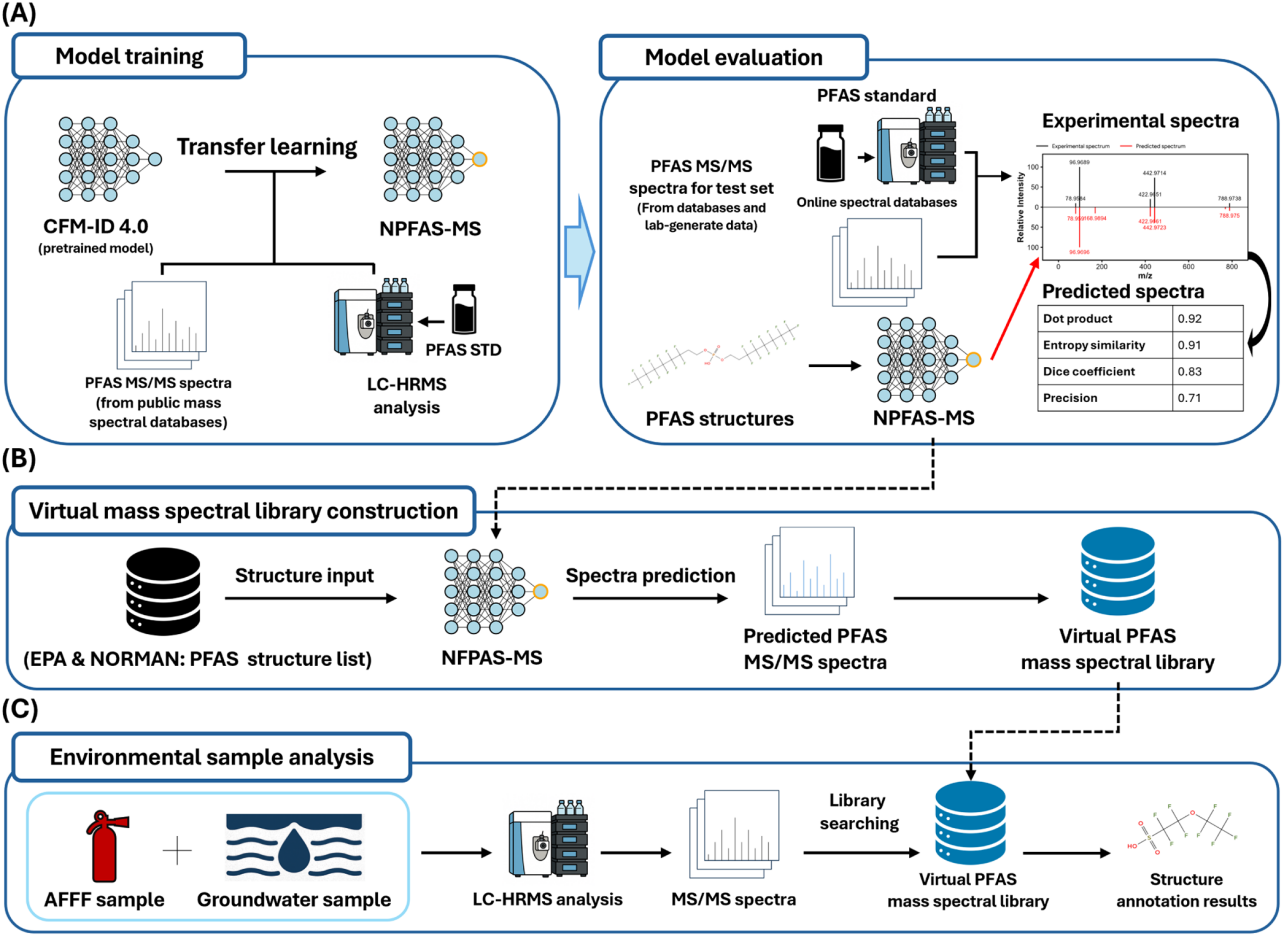
Overview of NPFAS-MS development and application workflow. (A) Model training and evaluation process. The pretrained model was fine-tuned through transfer learning with PFAS experimental MS/MS spectra to establish NPFAS-MS. Model evaluation involved comparing predicted PFAS spectra against PFAS experimental spectra analyzed by LC-HRMS and collected from public mass spectral databases, with performance assessed using various spectral similarity metrics. (B) Virtual PFAS mass spectral library construction. PFAS structures from the U.S. EPA and NORMAN databases were input into the NPFAS-MS model to generate predicted MS/MS spectra, creating a comprehensive virtual PFAS spectral library. (C) Environmental sample analysis workflow. AFFF and groundwater samples were analyzed by LC-HRMS to obtain MS/MS spectra, which were then searched against the virtual PFAS mass spectral library to characterize potential PFAS.

### NPFAS-MS Enhanced PFAS MS/MS Spectra Prediction

To enhance the predictive capability of NPFAS-MS for PFAS MS/MS spectra, a transfer learning strategy was adopted to fine-tune the pretrained model. For this purpose, as LC-HRMS-based nontargeted PFAS analysis primarily employs negative ESI mode,^41^ a data set containing 140 PFAS structures with corresponding 415 MS/MS spectra acquired in negative ESI mode across three different CE levels was collected. This data set comprised spectra from in-house analysis of reference standards and curated spectra from online public mass spectral databases. The data set was then classified into six categories based on different functional groups: per/polyfluoroalkyl carboxylic acids (PFCAs), per/polyfluoroalkyl sulfonic acids (PFSAs), per/polyfluoroalkane sulfonamides (PFSMs), per/polyfluoroalkyl phosphates (PFAPs), fluorotelomers (FTs), and Others.^42^ Subsequently, each category was randomly split into a training set and a hold-out test set with an approximate 70:30 ratio based on PFAS structures (except for three PFAS structures from online public databases that could not be separated into three CE levels after MS-Clustering and were directly assigned to the hold-out test set). This resulted in a training set of 98 structures with 294 spectra and a hold-out test set of 42 structures with 121 spectra (Figure S3). To further confirm the structural independence between the training and hold-out test sets, Extended-Connectivity Fingerprints with a radius of 3 (ECFP_6) were employed to calculate the Tanimoto similarity between the two data sets. The mean Tanimoto coefficient was 0.23 ± 0.13 (mean ± SD), demonstrating that the test set maintains reasonable structural independence from the training set (Figure S4).^43,44^ The training set was used to fine-tune the pretrained model to complete the construction of NPFAS-MS, while the hold-out test set was not used for model training but rather for evaluating the predictive capability of different spectral prediction models for PFAS. This strategy ensured that NPFAS-MS was trained and evaluated across different structural types. Following data preparation, a transfer learning strategy was adopted to fine-tune the pretrained CFM-ID 4.0 model, which can predict high-resolution negative ESI MS/MS spectra. Specifically, adopting the strategy proposed by Fei Wang et al.,^29^ all neural network parameters were frozen except for the last layer, which remained trainable. The model was then fine-tuned using the training set (Figure S5).

To evaluate the C2MS performance of NPFAS-MS, the similarity between predicted spectra and experimental spectra of known PFAS (hold-out test set) was assessed using four different spectral similarity metrics. These four metrics include dot product, which is commonly used in reference mass spectral database searches, as well as entropy similarity, dice coefficient, and precision. Additionally, the performance of NPFAS-MS was compared with three other spectral prediction models, including FIORA OS v0.1.0,^26^ PFAScreeneR,^24^ and CFM-ID 4.0, the pretrained model of NPFAS-MS. In the overall analysis across three CE levels, the other three models, which were not fine-tuned using PFAS MS/MS spectra, showed similar performance on dot product and entropy similarity-two metrics that evaluate both *m*/*z* and intensity. In contrast, NPFAS-MS demonstrated significantly improved performance compared to the other three spectral prediction models across all four metrics in the overall analysis (Figure 2). Similar performance trends were observed at individual CE levels (low, medium, and high). Specifically, in the overall analysis, relative to CFM-ID 4.0, NPFAS-MS improved from 0.37 to 0.56 (51% improvement) on dot product and from 0.30 to 0.51 (70% improvement) on entropy similarity, and from 0.21 to 0.42 (100% improvement) on dice coefficient, thereby demonstrating the effectiveness of the transfer learning strategy. For precision, NPFAS-MS improved from 0.28 to 0.57 compared to CFM-ID 4.0, and from 0.13 and 0.18 to 0.57 compared to FIORA and PFAScreeneR, respectively. The precision performance improved by over 100% relative to the other three models, indicating that NPFAS-MS is more capable of accurately predicting the fragment ions from PFAS.

**Figure 2:**
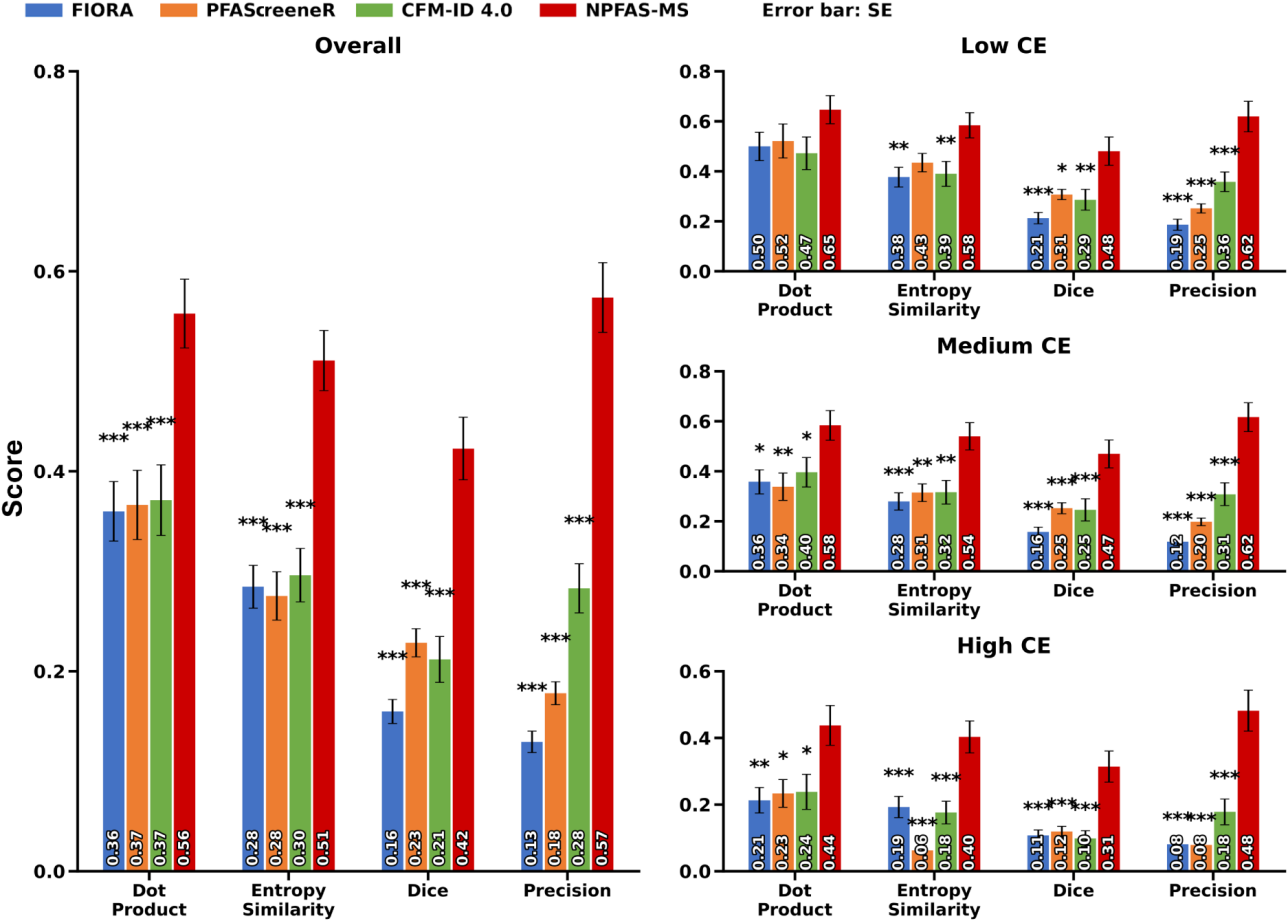
C2MS performance evaluation of NPFAS-MS against three spectral prediction models (FIORA, PFAScreeneR, and the pretrained model CFM-ID 4.0) in the hold-out test set using four similarity metrics (dot product, entropy similarity, dice coefficient, and precision) at different CE levels (overall, low CE, medium CE, and high CE). Bars represent mean scores, and error bars represent standard error (SE). Statistical significance with NPFAS-MS as the reference group (*p < 0.05, **p < 0.01, ***p < 0.001).

Additionally, representative experimental spectra and predicted spectra from four different models were shown for two PFAS from different sources in the hold-out test set. The experimental spectra of bis­[2-(perfluorohexyl)­ethyl] phosphate (6:2 diPAP) were acquired from reference standard analysis. At medium and high CE levels, the major product ions of 6:2 diPAP were *m*/*z* 96.9689 and *m*/*z* 78.9585. However, only CFM-ID 4.0 and NPFAS-MS successfully predicted these fragments, and the intensities predicted by NPFAS-MS better matched the experimental spectrum (Figure 3A). The experimental spectra of perfluoro­(2,5,8,11,14-pentamethyl-3,6,9,12,15-pentaoxaoctadecanoic) acid (HPFO-HxA) were collected from public mass spectral databases. At a low CE level, only NPFAS-MS accurately predicted its product ions, while the other three models primarily predicted the precursor ion (*m*/*z* 992.9091). At medium and high CE levels, only NPFAS-MS and PFAScreeneR accurately predicted the major product ions (*m*/*z* 184.9843, *m*/*z* 168.9894, and *m*/*z* 118.9927), with NPFAS-MS predicting intensities more consistent with the experimental spectrum and avoiding the prediction of nonexistent peaks (Figure 3B). Transfer learning strategies have been successfully employed in models such as DeepCDM^30^ and NPS-MS^29^ to accurately predict MS/MS spectra of chemically derived molecules and new psychoactive substances, respectively. Similarly, NPFAS-MS successfully utilized a transfer learning approach to enhance spectral prediction capability for PFAS. This improvement may be attributed not only to better reproduction of diagnostic ions, but also to an enhanced ability to capture fluorinated backbone-derived fragments characteristic of PFAS fragmentation, as illustrated by the representative examples in Figures 3 and S6. The more accurate prediction of these structurally informative ions likely enhances spectral specificity and contributes to the higher precision observed in Figure 2.

**Figure 3:**
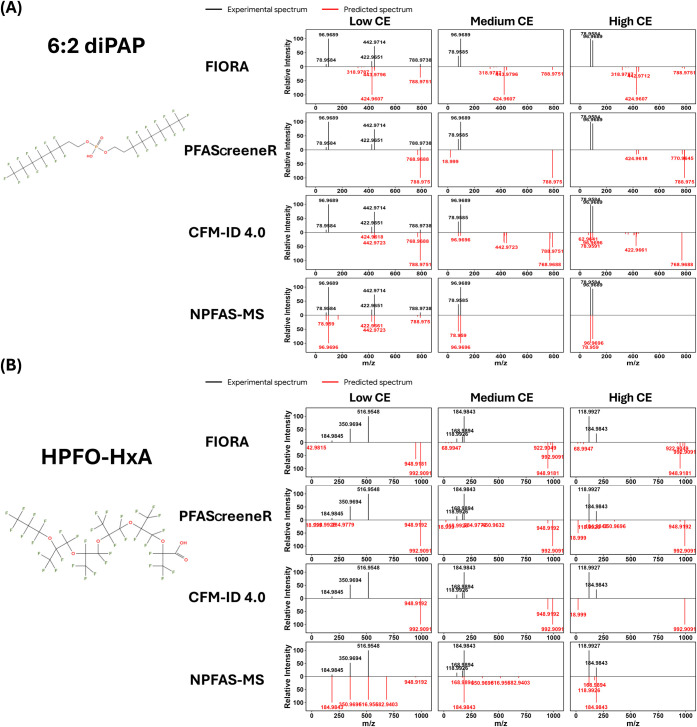
Experimental and predicted MS/MS spectra for two PFAS at different CE levels from the hold-out test set. (A) Spectra of 6:2 diPAP acquired from reference standard analysis. (B) Spectra of HPFO-HxA obtained from public mass spectral databases. For each compound, experimental spectra (black) and predicted spectra from four models (FIORA, PFAScreeneR, CFM-ID 4.0, and NPFAS-MS; red) are shown at low, medium, and high CE levels. Molecular structures are shown on the left.

Although the representative cases shown in Figure 3 highlight the improved predictive capability of NPFAS-MS, some PFAS still showed lower similarity to the experimental spectra. Analysis of representative examples suggests that these cases were not random but tended to fall into two broad categories (Figure S7). In some cases, the model correctly captured the major fragment ion but failed to reproduce additional minor experimental ions, resulting in an incomplete spectral match (Figure S7A). In other cases, the model predicted the major fragment correctly but also generated extra low-intensity peaks that were not observed experimentally, thereby reducing agreement with the measured spectrum (Figure S7B). While these representative examples do not encompass all failure modes, they highlight a current limitation of NPFAS-MS: incomplete prediction of lower-intensity fragment ions and imperfect fragment-intensity estimation can affect spectral similarity. As more PFAS MS/MS reference spectra become available, incorporating broader structural diversity into model training should further improve prediction completeness and fragment intensity accuracy.

To assess model stability and generalizability, five repeated random splits were performed using a stratified random 7:3 train/hold-out test split within each of the six structural categories.^30^ Performance across all five splits remained comparable under four spectral similarity metrics (dot product, entropy similarity, dice coefficient, and precision), with no statistically significant differences (*p* > 0.05 for all metrics; Figure S8). Additionally, UMAP visualization based on ECFP_6 fingerprints further showed that the training and hold-out test PFAS structures spanned multiple regions of the broader PFAS chemical space, encompassing 10,553 PFAS structures from U.S. EPA and NORMAN databases, rather than clustering in a single area (Figure S9). To further assess generalizability beyond the original hold-out test set, external validation was performed using two independent PFAS HRMS data sets, one comprising spectra acquired from authentic standard analyses,^45^ and the other comprising spectra acquired from environmental sample analyses.^46^ All PFAS structures represented in these external validation data sets were absent from the original training and hold-out test sets. NPFAS-MS showed comparable or better performance on the authentic standard-based data set, whereas lower similarity scores were observed for the environmental sample-based data set. The lower performance on the latter should be interpreted with caution because environmental sample-derived spectra may be influenced by sample complexity and coeluting compounds, and the confidence level of the reported annotations may differ from that of authentic standard-based spectra. In addition, some PFAS structures in the environmental sample-based data set may have fragmentation patterns that are less well represented in the current training data set (Figure S10).

### Virtual PFAS Mass Spectral Library Construction

Following model evaluation, a virtual mass spectral library was constructed using NPFAS-MS, and its mass spectrum-to-compound (MS2C) capability was assessed using the same hold-out test set. To construct a comprehensive virtual PFAS mass spectral library, PFAS structures were obtained from the U.S. EPA CompTox Chemicals Dashboard and NORMAN Substance Database,^22,47^ two leading regulatory databases for environmental contaminants, which collectively contained 20,391 PFAS structures of regulatory and environmental concern. This data set was systematically filtered by removing duplicated structures between the two databases, isotope-labeled compounds, precursor *m*/*z* > 1000, and structures containing metal cations in their SMILES. This curation process yielded a refined data set of 12,713 PFAS structures. Utilizing the CFM-ID 4.0 framework, NPFAS-MS identified ionizable structures, yielding a final set of 10,553 PFAS structures. For these structures, NPFAS-MS generated 31,659 predicted MS/MS spectra across three CE levels to establish a comprehensive virtual PFAS mass spectral library (Figure S11).

After establishing the virtual PFAS mass spectral library with NPFAS-MS, its MS2C performance was evaluated against three other virtual spectral libraries. Two in-house comparison libraries were generated from the same 10,553 PFAS structures using other two spectral prediction models (CFM-ID 4.0 and FIORA), with spectra predicted at the same three CE levels. The third comparison was made against the PFAScreeneR library,^27^ which included predicted spectra at three different CE levels for PFAS structures from the U.S. EPA database and in-house sources, as well as structures generated through *in silico* biotransformation. The MS2C capability of each virtual library was assessed through library searching against the same hold-out test set using dot product similarity. The most similar spectra from each library were retrieved and ranked to calculate top-*k* recall. The NPFAS-MS-based virtual mass spectral library demonstrated better annotation performance with 71.1% top-1 recall, representing a 15.7% improvement over the second-best virtual library generated by FIORA (Figure 4A). Furthermore, NPFAS-MS maintained robust performance across extended candidate lists, with 90.1% top-10 recall, which outperformed all comparison approaches (Figure 4B). Among the four virtual spectral libraries, 106 PFAS MS/MS spectra in the hold-out test set were successfully annotated with correct structures at top-1 recall. NPFAS-MS successfully annotated 81.1% of these spectra (Figure 4C). At top-5 recall, 116 spectra were correctly annotated, with NPFAS-MS successfully annotating 94% (Figure 4D). These results demonstrate that NPFAS-MS generated a more reliable virtual mass spectral library that enhanced PFAS annotation capabilities compared to other prediction approaches.

**Figure 4:**
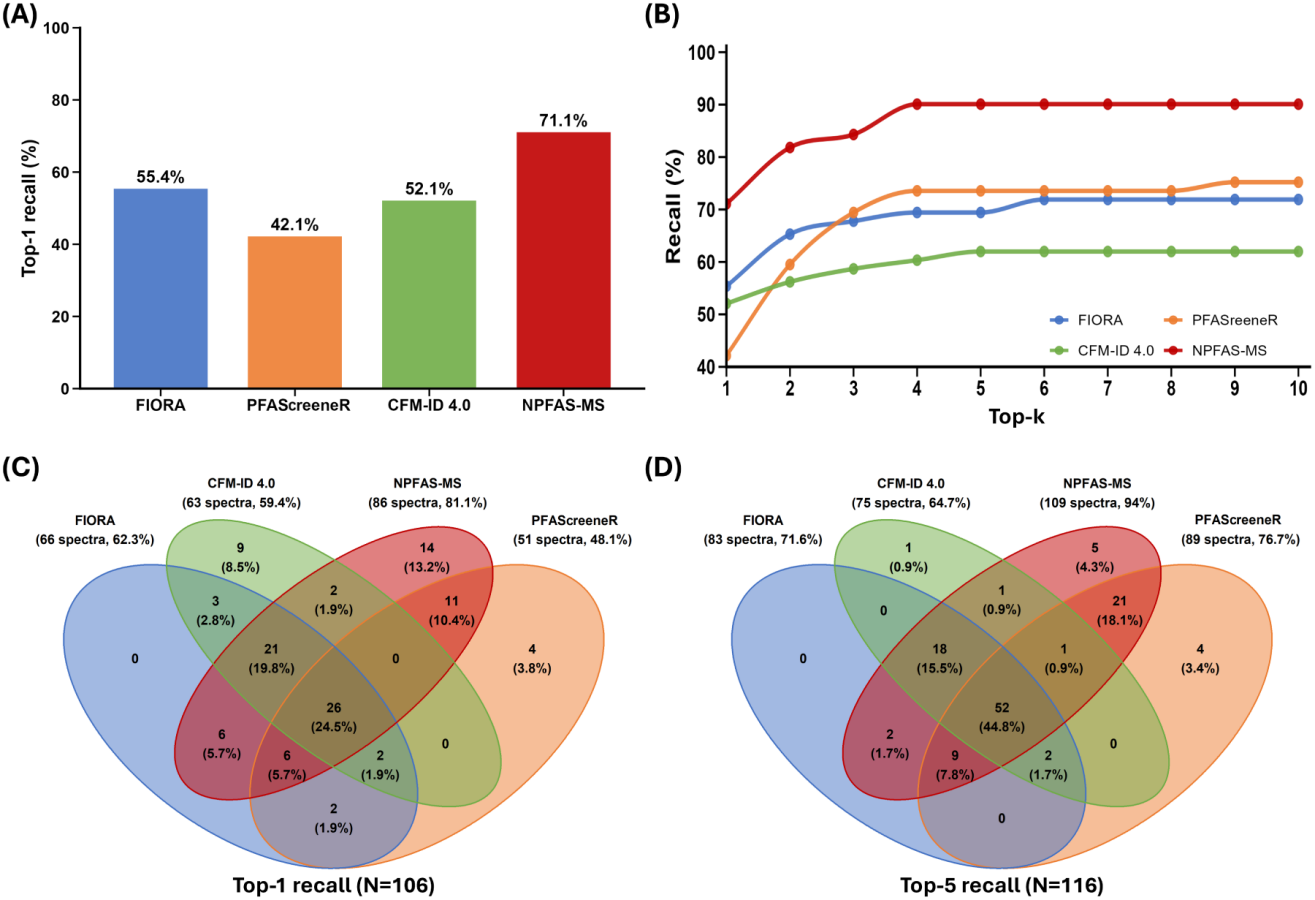
Library searching performance of four virtual PFAS mass spectral libraries. (A) Top-1 recall. (B) Recall performance across top-1 to top-10. (C) Distribution of successfully annotated spectra among the four libraries at top-1 recall (total *N* = 106). (D) Distribution at top-5 recall (total *N* = 116). All libraries were evaluated with the hold-out test set via dot product similarity searching. FIORA (blue), PFAScreeneR (orange), CFM-ID 4.0 (green), and NPFAS-MS (red).

### Environmental Application of NPFAS-MS-Generated Virtual Library for Nontargeted PFAS Analysis

Given the widespread application of PFAS across various industrial processes, improper handling and disposal practices have led to extensive environmental contamination, with discharged PFAS posing significant adverse health effects on both human populations and ecological systems. However, our knowledge of the full scope of PFAS present in environmental matrices remains limited. To maximize the detection of potentially existing PFAS in the environment, NPFAS-MS was applied to environmental sample analysis. According to previous environmental monitoring studies, AFFF-related and groundwater samples represent the two most frequently contaminated environmental sample types for PFAS detection. Therefore, eight commercial AFFF products were obtained from different manufacturers, and groundwater samples were collected from two different locations in northern Taiwan for PFAS analysis using the NPFAS-MS approach.

For nontargeted PFAS analysis in AFFF and groundwater samples, the relative abundance cutoff of 5% was applied to filter fragment ions in the MS/MS spectra. For MS/MS spectra searching against the virtual mass spectral library, precursor filtering with 5 ppm mass tolerance and a dot product threshold of >0.7 were applied for spectral matching (Figure S12),^38,48^ corresponding to level 3 (tentative candidate) of the confidence framework proposed by Schymanski et al.,^49^ indicating that candidate structures were proposed based on MS and MS/MS evidence. To evaluate the false positive rate (FPR) at different thresholds, a decoy data set was constructed using high-resolution MS/MS spectra with [M – H]^−^ adduct type from the MoNA, excluding all PFAS and their corresponding spectra. The decoy data set comprised 29,328 spectra from 5,840 non-PFAS compounds. At a dot product similarity threshold of 0.7, the FPR was 1.7%, which is lower than the FPR of 2.7% observed at a threshold of 0.5 and comparable to a previous study reporting an FPR of 2.4% at a threshold of 0.5 using 700 non-PFAS spectra (Figure S13).^50^ The threshold of 0.7 was therefore selected to reduce the FPR while maintaining sensitivity for detecting potential PFAS candidates. Additionally, the PFAS analysis performance of two different spectral databases was compared, including Compound Discoverer 3.3 (CD 3.3, Thermo Fisher Scientific) with mzCloud and PFAScreeneR.^27^ PFAScreeneR searches were performed in negative ion mode ([M – H]^−^) with 10 ppm mass tolerance and instrument type set as Orbitrap.

In the eight commercial AFFF products, 38 different PFAS were annotated through searching with the NPFAS-MS-generated virtual library (Table S5), while 18 different PFAS were annotated by Compound Discoverer with the mzCloud database, and 30 different PFAS were annotated by PFAScreeneR. The primary reference mass spectral database, mzCloud, a commercial database consisting mainly of MS/MS spectra acquired from authentic standards, showed 94% overlap of annotated PFAS with the virtual library generated by NPFAS-MS. Additionally, 28% of mzCloud-annotated PFAS overlapped with those from PFAScreeneR (Figure 5A). In addition to different spectral prediction approaches, the different types of predicted PFAS spectra included in PFAScreeneR may also contribute to this discrepancy, as PFAScreeneR primarily contains many spectra predicted from structures generated through biotransformation of PFAS.

**Figure 5:**
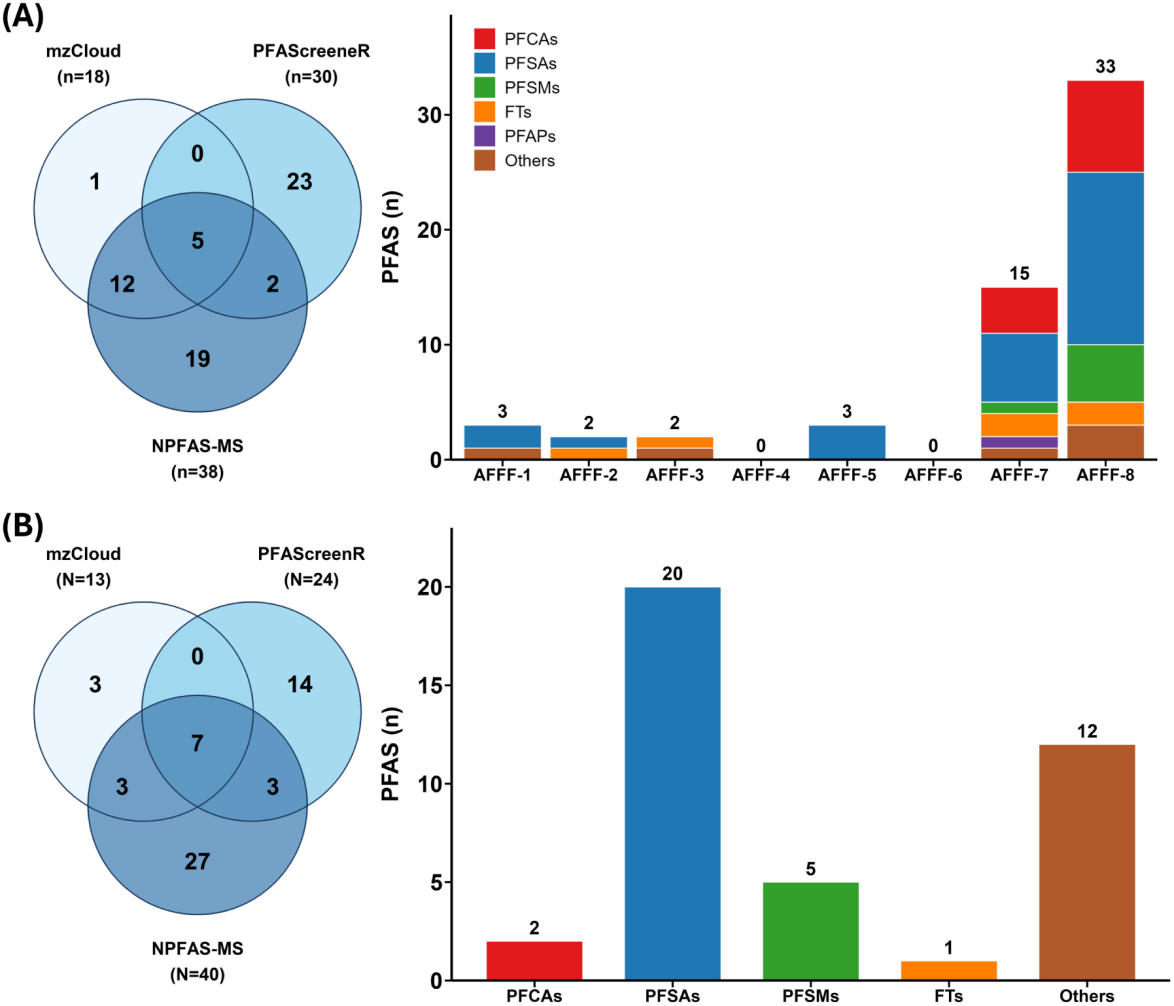
Comparison of PFAS annotation performance across different mass spectral databases in environmental samples. (A) AFFF sample analysis. The Venn diagram comparing PFAS annotations across three databases: NPFAS-MS-generated virtual library (*n* = 38), Compound Discoverer with mzCloud (*n* = 18), and PFAScreeneR (*n* = 30). The bar chart shows PFAS composition in each AFFF product (AFFF-1 through AFFF-8), by searching with the NPFAS-MS-generated virtual library. (B) Groundwater sample analysis. The Venn diagram comparing PFAS annotations across three databases: NPFAS-MS-generated virtual library (*n* = 40), Compound Discoverer with mzCloud (*n* = 13), and PFAScreeneR (*n* = 24). The bar chart displays the distribution of annotated PFAS by category using the NPFAS-MS-generated virtual library.

Based on the analysis results, the PFAS candidate composition in the eight commercial 3% AFFF products consisted predominantly of linear or branched PFSAs and PFCAs, as well as fluorotelomers (Figure 5A). This finding is consistent with AFFF manufacturing processes, as AFFFs can be categorized as either electrochemical fluorination (ECF)-based or FT-based formulations,^51^ with the latter utilizing fluorotelomer-derived surfactants as primary active ingredients. PFOS or perfluorohexanesulfonic acid (PFHxS) has been detected in multiple AFFF products, consistent with the presence of ECF-based formulations. This finding aligns with historical AFFF compositions, exemplified by products such as 3M Lightwater, which was adopted by the National Fire Protection Association and represents a typical ECF-based AFFF.^52,53^ In contrast, fluorotelomers were also characterized in multiple AFFF products, including 6:2 fluorotelomer sulfonic acid (FTS) and 8:2 FTS. Additionally, N-(3-(dimethylamino)­propyl)­pentadecafluoro-1-heptanesulfonamide and 7H-PFOS were annotated, as byproducts commonly associated with AFFF formulations (Figure S14).^1^ In the AFFF samples, searching with the NPFAS-MS-generated virtual library enabled the annotation of (E)-5H-perfluorooct-6-ene-1-sulfonic acid (H-UPFOS), a hydrogen-substituted unsaturated PFSA that is not included in online public spectral databases (Figure 6A). Furthermore, for successfully matched fragments (*m*/*z* 422.9369, 292.9826), NPFAS-MS was employed to perform candidate structure annotation of the corresponding product ions. The remaining unmatched major product ions were annotated by submitting the NPFAS-MS-assigned molecular formula and query spectra to MetFrag for possible structure elucidation (Figure S15).^54^ Given that AFFF applications at fire training sites and emergency response scenarios often result in direct environmental discharge, these findings indicate that current commercial AFFF products could potentially contribute as ongoing sources of diverse PFAS contamination to environmental systems.^51,55,56^

**Figure 6:**
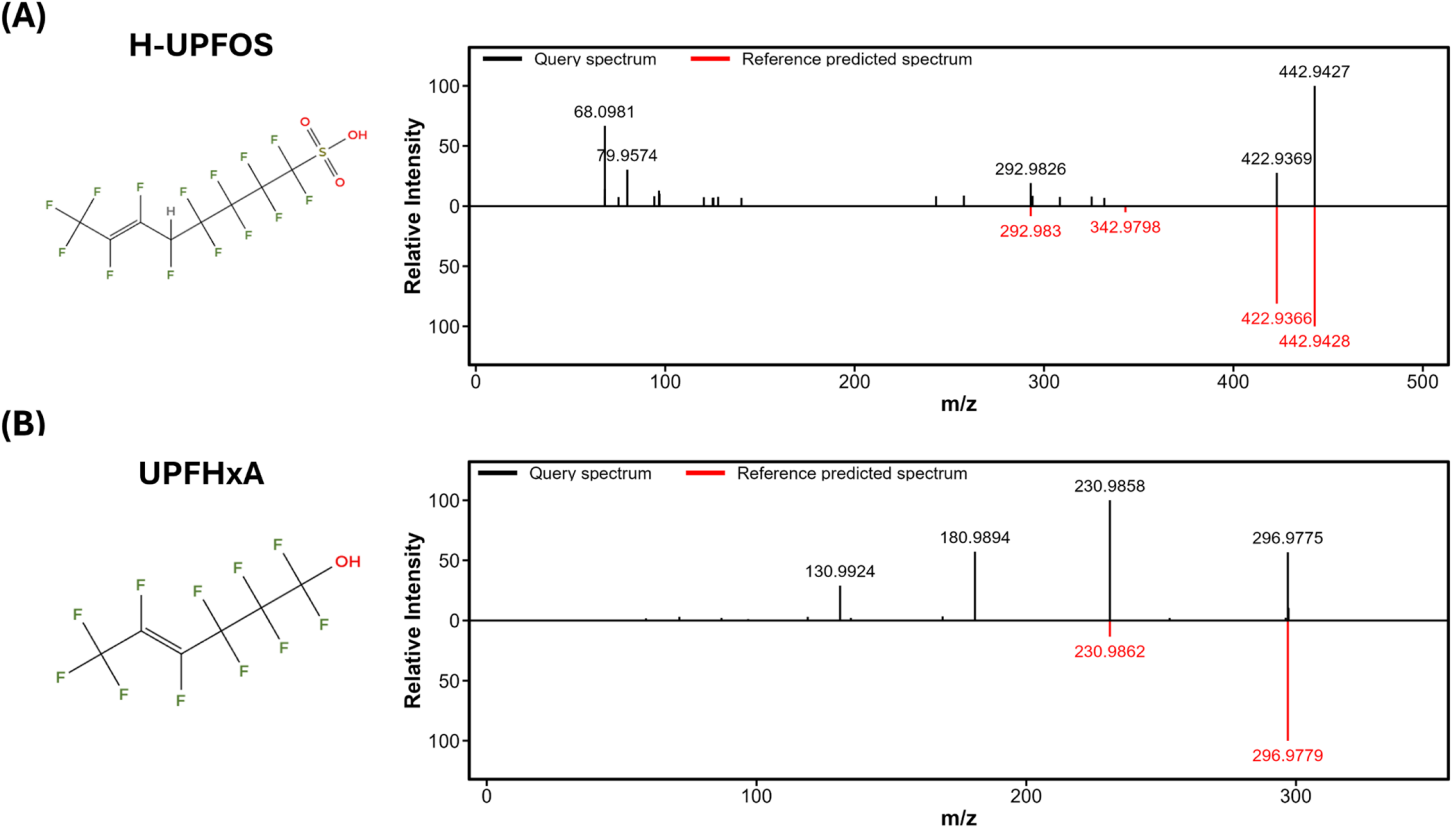
Annotation of emerging PFAS in environmental samples using NPFAS-MS-generated virtual library. (A) Query spectrum (black) and NPFAS-MS predicted spectrum (red) for H-UPFOS, a hydrogen-substituted unsaturated PFSA annotated in AFFF products (dot product score: 0.92). The precursor ion ([M – H]^−^, *m*/*z* 442.9427) and major product ions (*m*/*z* 422.9369, 292.9826, 79.9574, 68.0981) are labeled. (B) Query spectrum (black) and NPFAS-MS predicted spectrum (red) for UPFHxA, an unsaturated perfluorinated alcohol annotated in groundwater samples (dot product score: 0.78). Molecular structure is shown on the left. The precursor ion ([M – H]^−^, *m*/*z* 296.9775) and major product ions (*m*/*z* 230.9858, 180.9894, 130.9924) are labeled.

In the pooled groundwater samples, 40 different PFAS were annotated through searching with the NPFAS-MS-generated virtual library, while 13 different PFAS were annotated by Compound Discoverer with the mzCloud database, and 24 different PFAS were annotated by PFAScreeneR. Compound Discoverer with the mzCloud showed 76.9% overlap of annotated PFAS with the virtual library generated by NPFAS-MS. Additionally, 54% of Compound Discoverer-annotated PFAS overlapped with PFAS annotated using PFAScreeneR (Figure 5B).

The potential PFAS contained in groundwater samples were primarily PFSAs and PFSMs, with different types of PFAS also detected (Figure 5B). The searching results with the virtual library also indicated that groundwater samples contain emerging PFAS candidates. These include n:2 FTS, hydrogen-substituted perfluorosulfonic acids (H-PFSA), ultrashort-chain PFAS, and other emerging PFAS (Table S6). These annotated results were consistent with recent studies showing increasing detection of emerging PFAS in groundwater systems.^16,35^ The annotated PFAS candidates also included perfluoro-4-ethylcyclohexane (PFECHS), a cyclic PFSA that serves as a substitute for PFOS and is primarily applied as an erosion inhibitor in hydraulic fluids. Recent studies have also demonstrated the detection of PFECHS in fish and aquatic environments, indicating that short-term exposure may potentially lead to toxic effects (Figure S16A).^57,58^ The annotated results also included ultrashort-chain PFAS. With growing regulatory restrictions on long-chain PFAS, short- and ultrashort-chain PFAS are increasingly being adopted as alternatives (Figure S16B).^15^ Notably, through searching with the virtual library generated by NPFAS-MS, (E)-1,1,2,2,3,3,4,5,6,6,6-undecafluorohex-4-en-1-ol (UPFHxA), an unsaturated perfluorinated alcohol with a C6 carbon chain, was annotated (Figure 6B). Subsequent searches using the “Draw” function in SciFinder^n^ revealed that this compound had never been reported. However, an unsaturated perfluorinated alcohol with a C7 carbon chain has been previously detected in paired maternal and cord sera from pregnant women through diagnostic fragment-based approaches,^59^ indicating that knowledge of this class of PFAS remains limited. Similarly, NPFAS-MS was employed to annotate the fragment ions of successfully matched fragments. While NPFAS-MS successfully predicted major fragment ions (*m*/*z* 230.9858), two major product ions (*m*/*z* 180.9894, 130.9924) were not captured by the model. The remaining major product ions were therefore structurally elucidated by submitting the NPFAS-MS-assigned molecular formula and query spectra to MetFrag (Figure S17).

It should be noted that while NPFAS-MS enables comprehensive screening of potential PFAS in the environment, the virtual mass spectral library searching primarily serves as an effective prioritization tool. When reference standards are available, additional structural confirmation can be achieved. In particular, discrimination of closely related branched or positional PFAS isomers remains challenging when the distinguishing fragment ions are of low relative intensity. For additional confirmation, representative PFAS annotated in environmental samples, including compounds from the model training and hold-out test sets, were evaluated against authentic reference standards across different environmental matrices (Figure S18).

### Environmental Implications

As increasing numbers of emerging PFAS are being identified in diverse environmental samples (e.g., river, food packaging, and groundwater), toxicological studies have demonstrated that not only legacy PFAS like PFOA and PFOS, but also these emerging PFAS can cause significant adverse effects on organisms and ecosystems.^60,61^ For instance, exposure to fluorotelomer substances has been linked to developmental impacts in male infants,^62^ and short-term exposure to cyclic PFSAs may potentially lead to toxic effects,^58^ highlighting the urgent need for comprehensive PFAS monitoring. In this study, we developed NPFAS-MS, a transfer learning-based neural network model fine-tuned with curated PFAS spectra acquired at multiple CEs. Compared to other general spectral prediction models and existing spectral databases, NPFAS-MS demonstrated higher prediction accuracy and annotation efficiency. Using the NPFAS-MS approach, 38 PFAS candidates were successfully annotated in AFFF products and 40 in groundwater samples. Our analysis of AFFF products and groundwater samples revealed emerging and previously undetected PFAS. These findings indicate that both legacy and emerging PFAS continue to be released into the environment. Notably, the annotation results indicated that six out of the eight commercial AFFF products may still contain PFAS as active ingredients. Currently, regulations for emerging PFAS across countries remain unclear, and acquiring extensive reference standards for environmental PFAS identification presents substantial challenges. Therefore, rapid and large-scale screening of potential PFAS in contaminated or exposed environments has become critically important. The virtual PFAS mass spectral library generated by NPFAS-MS demonstrates that it can be applied across different environmental matrices and enables large-scale annotation of potential legacy and emerging PFAS. However, the practical coverage of the current virtual library remains limited to the 10,553 curated PFAS structures included in the database; PFAS absent from this structure set, including newly reported PFAS, transformation products, or biotransformation products, cannot be directly annotated through library searching. Future expansion of the structure library by incorporating newly reported PFAS and transformation products will further improve the coverage and applicability of NPFAS-MS. To enhance accessibility and practical application, we developed a user-friendly web-based tool that supports both direct MS/MS spectral prediction from user-input structures and spectral matching against the NPFAS-MS virtual library, enabling users to easily predict PFAS MS/MS spectra and annotate PFAS candidates through library searching. NPFAS-MS is designed to operate optimally under the following conditions: negative ESI ionization mode with [M – H]^−^ precursor ions with molecular mass ≤1000 Da, compatibility with both QTOF and Orbitrap acquisition platforms. All fragment *m*/*z* values in the database are theoretically calculated, and the tool allows users to define their own mass accuracy threshold to accommodate data from instruments with varying resolving power. Overall, NPFAS-MS provides an expandable virtual-library framework that supports comprehensive environmental monitoring and regulatory efforts aimed at addressing the rapidly evolving challenge of PFAS chemical diversity.

## Supplementary Material



## Notes

PFAS reference standards (Table S1); AFFF sample information and source details (Table S2); model configuration parameters for CFM-ID 4.0 (Table S3); model configuration parameters for NPFAS-MS (Table S4); PFAS candidates annotated across eight commercial AFFF products using the virtual library constructed with NPFAS-MS (Table S5); PFAS candidates annotated in groundwater samples using the virtual library constructed with NPFAS-MS (Table S6); workflow for MS/MS data acquisition and nontargeted analysis (Figure S1); representative spectra selection by MS-Clustering (Figure S2); training and hold-out test set distribution of PFAS structures and spectra (Figure S3); Tanimoto coefficient distribution between the training and hold-out test sets (Figure S4); transfer learning architecture of NPFAS-MS (Figure S5); representative examples illustrating the improved PFAS-specific fragmentation prediction of NPFAS-MS relative to the comparison models (Figure S6); representative cases illustrating two limitation patterns of NPFAS-MS spectral prediction (Figure S7); performance across five repeated random splits under four spectral similarity metrics (dot product, entropy similarity, Dice coefficient, and precision) (Figure S8); UMAP visualization of chemical space coverage for training and hold-out test PFAS (Figure S9); external validation of NPFAS-MS using two independent PFAS HRMS data sets (Figure S10); construction of a virtual PFAS mass spectral library (Figure S11); PFAS annotation performance at different dot product score thresholds in environmental samples (Figure S12); false positive rate of different dot product similarity thresholds using non-PFAS decoy data set (Figure S13); byproduct PFAS detected in commercial AFFF products (Figure S14); structural annotation of (E)-5H-perfluorooct-6-ene-1-sulfonic acid (H-UPFOS) in AFFF products (Figure S15); representative emerging PFAS detected in groundwater samples (Figure S16); structural annotation of (E)-1,1,2,2,3,3,4,5,6,6,6-undecafluorohex-4-en-1-ol (UPFHxA) (Figure S17); validation of NPFAS-MS predicted spectra against reference standards across different environmental matrices (Figure S18) (PDF)

## References

[1] Barzen-Hanson K. A., Roberts S. C., Choyke S., Oetjen K., McAlees A., Riddell N., McCrindle R., Ferguson P. L., Higgins C. P., Field J. A. (2017). Discovery of 40 Classes of Per- and Polyfluoroalkyl
Substances in Historical Aqueous Film-Forming Foams (AFFFs) and AFFF-Impacted
Groundwater. Environ. Sci. Technol.

[2] Gluge J., Scheringer M., Cousins I. T., DeWitt J. C., Goldenman G., Herzke D., Lohmann R., Ng C. A., Trier X., Wang Z. (2020). An overview of the uses of per- and polyfluoroalkyl substances (PFAS). Environ. Sci.: Processes Impacts.

[3] Evich M. G., Davis M. J. B., McCord J. P., Acrey B., Awkerman J. A., Knappe D. R. U., Lindstrom A. B., Speth T. F., Tebes-Stevens C., Strynar M. J., Wang Z., Weber E. J., Henderson W. M., Washington J. W. (2022). Per- and
polyfluoroalkyl substances in the environment. Science.

[4] Gao C., Drage D. S., Abdallah M. A.-E., Quan F., Zhang K., Hu S., Zhao X., Zheng Y., Harrad S., Qiu W. (2024). Factors Influencing
Concentrations of PFAS in Drinking Water: Implications for Human Exposure. ACS EST Water.

[5] Gebbink W. A., van Asseldonk L., van Leeuwen S. P. J. (2017). Presence of Emerging Per- and Polyfluoroalkyl
Substances (PFASs) in River and Drinking Water near a Fluorochemical
Production Plant in the Netherlands. Environ.
Sci. Technol.

[6] Kurwadkar S., Dane J., Kanel S. R., Nadagouda M. N., Cawdrey R. W., Ambade B., Struckhoff G. C., Wilkin R. (2022). Per- and polyfluoroalkyl substances in water and wastewater:
A critical review of their global occurrence and distribution. Sci. Total Environ.

[7] Zhou T., Li X., Liu H., Dong S., Zhang Z., Wang Z., Li J., Nghiem L. D., Khan S. J., Wang Q. (2024). Occurrence, fate, and
remediation for per-and polyfluoroalkyl substances (PFAS) in sewage
sludge: A comprehensive review. J. Hazard. Mater.

[8] Li Y., Yao J., Zhang J., Pan Y., Dai J., Ji C., Tang J. (2022). First Report on the
Bioaccumulation and Trophic Transfer of Perfluoroalkyl
Ether Carboxylic Acids in Estuarine Food Web. Environ. Sci. Technol.

[9] Wang L., Yang T., Liu X., Liu J., Liu W. (2024). Critical Evaluation
and Meta-Analysis of Ecotoxicological Data on Per- and Polyfluoroalkyl
Substances (PFAS) in Freshwater Species. Environ.
Sci. Technol.

[10] Domingo J. L., Nadal M. (2019). Human exposure to per- and polyfluoroalkyl substances (PFAS) through
drinking water: A review of the recent scientific literature. Environ. Res.

[11] Gaballah S., Swank A., Sobus J. R., Howey X. M., Schmid J., Catron T., McCord J., Hines E., Strynar M., Tal T. (2020). Evaluation of Developmental Toxicity, Developmental Neurotoxicity,
and Tissue Dose in Zebrafish Exposed to GenX and Other PFAS. Environ. Health Perspect.

[12] Roth K., Yang Z., Agarwal M., Liu W., Peng Z., Long Z., Birbeck J., Westrick J., Liu W., Petriello M. C. (2021). Exposure to a mixture of legacy, alternative, and replacement
per- and polyfluoroalkyl substances (PFAS) results in sex-dependent
modulation of cholesterol metabolism and liver injury. Environ. Int.

[13] Zheng G., Schreder E., Dempsey J. C., Uding N., Chu V., Andres G., Sathyanarayana S., Salamova A. (2021). Per- and Polyfluoroalkyl
Substances (PFAS) in Breast Milk: Concerning Trends for Current-Use
PFAS. Environ. Sci. Technol.

[14] Golden-Mason L., Salomon M. P., Matsuba C., Wang Y., Setiawan V. W., Chatzi L., Maretti-Mira A. C. (2025). Assessing
the impact of perfluoroalkyl
substances on liver health: A comprehensive study using multi-donor
human liver spheroids. Environ. Int.

[15] Ateia M., Maroli A., Tharayil N., Karanfil T. (2019). The overlooked short-
and ultrashort-chain poly- and perfluorinated substances: A review. Chemosphere.

[16] Ghorbani
Gorji S., Gomez Ramos M. J., Dewapriya P., Schulze B., Mackie R., Nguyen T. M. H., Higgins C. P., Bowles K., Mueller J. F., Thomas K. V., Kaserzon S. L. (2024). New PFASs
Identified in AFFF Impacted Groundwater by Passive Sampling and Nontarget
Analysis. Environ. Sci. Technol.

[17] Chen Y. J., Wang R. D., Shih Y. L., Chin H. Y., Lin A. Y. (2024). Emerging
Perfluorobutane Sulfonamido Derivatives as a New Trend of Surfactants
Used in the Semiconductor Industry. Environ.
Sci. Technol.

[18] Chen Y. C., Hsu J. F., Chang C. W., Li S. W., Yang Y. C., Chao M. R., Chen H. C., Liao P. C. (2023). Connecting chemical
exposome to human health using high-resolution mass spectrometry-based
biomonitoring: Recent advances and future perspectives. Mass Spectrom. Rev.

[19] Lai Y., Koelmel J. P., Walker D. I., Price E. J., Papazian S., Manz K. E., Castilla-Fernandez D., Bowden J. A., Nikiforov V., David A., Bessonneau V., Amer B., Seethapathy S., Hu X., Lin E. Z., Jbebli A., McNeil B. R., Barupal D., Cerasa M., Xie H., Kalia V., Nandakumar R., Singh R., Tian Z., Gao P., Zhao Y., Froment J., Rostkowski P., Dubey S., Coufalikova K., Selicova H., Hecht H., Liu S., Udhani H. H., Restituito S., Tchou-Wong K. M., Lu K., Martin J. W., Warth B., Godri Pollitt K. J., Klanova J., Fiehn O., Metz T. O., Pennell K. D., Jones D. P., Miller G. W. (2024). High-Resolution
Mass Spectrometry for Human Exposomics: Expanding Chemical Space Coverage. Environ. Sci. Technol.

[20] Vinaixa M., Schymanski E. L., Neumann S., Navarro M., Salek R. M., Yanes O. (2016). Mass spectral
databases for LC/MS- and GC/MS-based metabolomics:
State of the field and future prospects. TrAC,
Trends Anal. Chem.

[21] mzCloud. Advanced Mass Spectral Database, 2025. https://www.mzcloud.org/.

[22] Williams A. J., Grulke C. M., Edwards J., McEachran A. D., Mansouri K., Baker N. C., Patlewicz G., Shah I., Wambaugh J. F., Judson R. S., Richard A. M. (2017). The CompTox
Chemistry Dashboard: A community data resource for environmental chemistry. J. Cheminf.

[23] Beck A. G., Muhoberac M., Randolph C. E., Beveridge C. H., Wijewardhane P. R., Kenttamaa H. I., Chopra G. (2024). Recent Developments
in Machine Learning for Mass Spectrometry. ACS
Meas. Sci. Au.

[24] Wang F., Liigand J., Tian S., Arndt D., Greiner R., Wishart D. S. (2021). CFM-ID 4.0: More
Accurate ESI-MS/MS Spectral Prediction
and Compound Identification. Anal. Chem.

[25] Wei J. N., Belanger D., Adams R. P., Sculley D. (2019). Rapid Prediction of
Electron-Ionization Mass Spectrometry Using Neural Networks. ACS Cent. Sci.

[26] Nowatzky Y., Russo F. F., Lisec J., Kister A., Reinert K., Muth T., Benner P. F. (2025). Local neighborhood-based
prediction
of compound mass spectra from single fragmentation events. Nat. Commun.

[27] Getzinger G. J., Higgins C. P., Ferguson P. L. (2021). Structure Database
and In Silico
Spectral Library for Comprehensive Suspect Screening of Per- and Polyfluoroalkyl
Substances (PFASs) in Environmental Media by High-resolution Mass
Spectrometry. Anal. Chem.

[28] Pan S. J., Yang Q. (2010). A Survey on Transfer
Learning. IEEE Trans.
Knowl. Data Eng.

[29] Wang F., Pasin D., Skinnider M. A., Liigand J., Kleis J. N., Brown D., Oler E., Sajed T., Gautam V., Harrison S., Greiner R., Foster L. J., Dalsgaard P. W., Wishart D. S. (2023). Deep Learning-Enabled
MS/MS Spectrum Prediction Facilitates
Automated Identification Of Novel Psychoactive Substances. Anal. Chem.

[30] Chen B., Li H., Huang R., Tang Y., Li F. (2024). Deep learning prediction
of electrospray ionization tandem mass spectra of chemically derived
molecules. Nat. Commun.

[31] Dlugogorski B. Z., Schaefer T. H. (2021). Compatibility of
aqueous film-forming foams (AFFF)
with sea water. Fire Safety J.

[32] U.S. Environmental Protection Agency (EPA). Method 1633, Revision A Analysis of Per- and Polyfluoroalkyl Substances (PFAS) in Aqueous, Solid, Biosolids, and Tissue Samples by LC-MS/MS, EPA, 2025.

[33] Tsugawa H., Cajka T., Kind T., Ma Y., Higgins B., Ikeda K., Kanazawa M., VanderGheynst J., Fiehn O., Arita M. (2015). MS-DIAL: Data-independent MS/MS deconvolution
for comprehensive metabolome analysis. Nat.
Methods.

[34] Bugsel B., Zwiener C. (2020). LC-MS screening of poly- and perfluoroalkyl substances
in contaminated soil by Kendrick mass analysis. Anal. Bioanal. Chem.

[35] Sun J., Liu Y., Yao L., Guo Y., Ma C., Xiang T., Cheng Z., Deng Y., Xie X., Qu G., Shi J., Jiang G., Wang Y. (2025). Suspect and
Nontarget Analysis of
Per- and Polyfluoroalkyl Substances in Groundwater Underlying Different
Land-Use Areas. Environ. Sci. Technol.

[36] MassBank of North America. 2025. https://mona.fiehnlab.ucdavis.edu/.

[37] Ragland J. M., Place B. J. (2024). A Portable and Reusable
Database Infrastructure for
Mass Spectrometry, and Its Associated Toolkit (The DIMSpec Project). J. Am. Soc. Mass Spectrom.

[38] Horai H., Arita M., Kanaya S., Nihei Y., Ikeda T., Suwa K., Ojima Y., Tanaka K., Tanaka S., Aoshima K., Oda Y., Kakazu Y., Kusano M., Tohge T., Matsuda F., Sawada Y., Hirai M. Y., Nakanishi H., Ikeda K., Akimoto N., Maoka T., Takahashi H., Ara T., Sakurai N., Suzuki H., Shibata D., Neumann S., Iida T., Tanaka K., Funatsu K., Matsuura F., Soga T., Taguchi R., Saito K., Nishioka T. (2010). MassBank:
A public repository for
sharing mass spectral data for life sciences. J. Mass Spectrom.

[39] Frank A. M., Bandeira N., Shen Z., Tanner S., Briggs S. P., Smith R. D., Pevzner P. A. (2008). Clustering Millions of Tandem Mass
Spectra. J. Proteome Res.

[40] Li Y., Kind T., Folz J., Vaniya A., Mehta S. S., Fiehn O. (2021). Spectral entropy outperforms
MS/MS dot product similarity for small-molecule
compound identification. Nat. Methods.

[41] Megson D., Bruce-Vanderpuije P., Idowu I. G., Ekpe O. D., Sandau C. D. (2025). A systematic
review for non-targeted analysis of per- and polyfluoroalkyl substances
(PFAS). Sci. Total Environ.

[42] Liu Y., D’Agostino L. A., Qu G., Jiang G., Martin J. W. (2019). High-resolution
mass spectrometry (HRMS) methods for nontarget discovery and characterization
of poly- and per-fluoroalkyl substances (PFASs) in environmental and
human samples. TrAC, Trends Anal. Chem.

[43] Patterson D. E., Cramer R. D., Ferguson A. M., Clark R. D., Weinberger L. E. (1996). Neighborhood
Behavior: A Useful Concept for Validation of “Molecular Diversity”
Descriptors. J. Med. Chem.

[44] Schur C., Gasser L., Perez-Cruz F., Schirmer K., Baity-Jesi M. (2023). A benchmark
dataset for machine learning in ecotoxicology. Sci. Data.

[45] Wang H., Kuo T.-C., Tseng Y. J. (2025). DeePFAS: Deep-Learning-Enabled Rapid
Annotation of PFAS: Enhancing Nontargeted Screening through Spectral
Encoding and Latent Space Analysis. Environ.
Sci. Technol.

[46] Jiao Z., Taniyasu S., Yu N., Wang X., Yamashita N., Wei S. (2025). Two-layer homolog network approach for PFAS nontarget screening and
retrospective data mining. Nat. Commun.

[47] NORMAN Substance Database – NORMAN SusDat. 2025. https://www.norman-network.com/nds/susdat/.

[48] Huan T., Tang C., Li R., Shi Y., Lin G., Li L. (2015). MyCompoundID MS/MS Search: Metabolite
Identification Using a Library
of Predicted Fragment-Ion-Spectra of 383,830 Possible Human Metabolites. Anal. Chem.

[49] Schymanski E. L., Jeon J., Gulde R., Fenner K., Ruff M., Singer H. P., Hollender J. (2014). Identifying
small molecules via high
resolution mass spectrometry: Communicating confidence. Environ. Sci. Technol.

[50] Wang X., Yu N., Jiao Z., Li L., Yu H., Wei S. (2024). Machine learning–enhanced
molecular network reveals global exposure to hundreds of unknown PFAS. Sci. Adv.

[51] Yan P. F., Dong S., Pennell K. D., Capiro N. L. (2024). A review of the
occurrence and microbial transformation of per- and polyfluoroalkyl
substances (PFAS) in aqueous film-forming foam (AFFF)-impacted environments. Sci. Total Environ.

[52] Rotander A., Toms L. M., Aylward L., Kay M., Mueller J. F. (2015). Elevated
levels of PFOS and PFHxS in firefighters exposed to aqueous film forming
foam (AFFF). Environ. Int.

[53] Baduel C., Mueller J. F., Rotander A., Corfield J., Gomez-Ramos M. J. (2017). Discovery
of novel per- and polyfluoroalkyl substances (PFASs) at a fire fighting
training ground and preliminary investigation of their fate and mobility. Chemosphere.

[54] Ruttkies C., Schymanski E. L., Wolf S., Hollender J., Neumann S. (2016). MetFrag relaunched:
Incorporating strategies beyond
in silico fragmentation. J. Cheminform.

[55] Douglas G. B., Vanderzalm J. L., Williams M., Kirby J. K., Kookana R. S., Bastow T. P., Bauer M., Bowles K. C., Skuse D., Davis G. B. (2023). PFAS contaminated
asphalt and concrete-Knowledge gaps
for future research and management. Sci. Total
Environ.

[56] Wanzek T. A., Field J. A., Kostarelos K. (2024). Repeated Aqueous
Film-Forming Foams
Applications: Impacts on Polyfluoroalkyl Substances Retention in Saturated
Soil. Environ. Sci. Technol.

[57] De
Silva A. O., Spencer C., Scott B. F., Backus S., Muir D. C. (2011). Detection of a cyclic perfluorinated acid, perfluoroethylcyclohexane
sulfonate, in the Great Lakes of North America. Environ. Sci. Technol.

[58] Mahoney H., Ankley P., Roberts C., Lamb A., Schultz M., Zhou Y., Giesy J. P., Brinkmann M. (2024). Unveiling
the Molecular Effects of Replacement and Legacy PFASs: Transcriptomic
Analysis of Zebrafish Embryos Reveals Surprising Similarities and
Potencies. Environ. Sci. Technol.

[59] Li Y., Yu N., Du L., Shi W., Yu H., Song M., Wei S. (2020). Transplacental
Transfer of Per- and Polyfluoroalkyl Substances Identified
in Paired Maternal and Cord Sera Using Suspect and Nontarget Screening. Environ. Sci. Technol.

[60] Sunderland E. M., Hu X. C., Dassuncao C., Tokranov A. K., Wagner C. C., Allen J. G. (2019). A review of the
pathways of human exposure to poly-
and perfluoroalkyl substances (PFASs) and present understanding of
health effects. J. Exposure Sci. Environ. Epidemiol.

[61] Lohmann R., Cousins I. T., DeWitt J. C., Gluge J., Goldenman G., Herzke D., Lindstrom A. B., Miller M. F., Ng C. A., Patton S., Scheringer M., Trier X., Wang Z. (2020). Are Fluoropolymers
Really of Low Concern for Human and Environmental Health and Separate
from Other PFAS?. Environ. Sci. Technol.

[62] Wu M., Wu Y., Guo W., Xi J., Miao M., Zhao H., Liang H., Xue J., Zhu H., Sun H. (2025). Prenatal exposure
to legacy and emerging PFAS and sex-specific associations with fetal
growth: Evidence from a Chinese birth cohort. Environ. Res.

