# Supplementary material for: Enhanced Environmental PFAS Characterization Using a Virtual High-Resolution Mass Spectral Library Generated by Transfer Learning-Based Neural Network
Source: Environ Sci Technol. 2026 Jun 10;60(24):17341–54. doi: 10.1021/acs.est.6c05733 (PMC13296486; doi:10.1021/acs.est.6c05733)
Supplement: Supplementary file 1 [file es6c05733_si_001.pdf]

Supporting Information

Enhanced Environmental PFAS Characterization Using a Virtual High-Resolution Mass Spectral Library Generated by Transfer Learning-Based Neural Network

Yi-Chi Chen[1], Hsin-Yi Wu[2], Man-Ni Zhuang[1], Chen-Ming Yi[3], Wei-Sheng Wu[4,], and Pao-Chi Liao[1,*]*

1. Department of Environmental and Occupational Health, College of Medicine, National Cheng Kung University, Tainan 704, Taiwan

2. Instrumentation Center, National Taiwan University, Taipei 106, Taiwan

3. Institute of Computer and Communication Engineering, National Cheng Kung University, Tainan 701, Taiwan

4. Department of Electrical Engineering, National Cheng Kung University, Tainan 701, Taiwan

*Correspondences:

Dr. Wei-Sheng Wu

Department of Electrical Engineering, National Cheng Kung University

E-mail: wessonwu@mail.ncku.edu.tw

Dr. Pao-Chi Liao

Department of Environmental and Occupational Health, College of Medicine, National Cheng Kung University

E-mail: liaopc@mail.ncku.edu.tw

Summary: 6 tables, 18 figures, 33 pages

Table of Contents

Table S1. PFAS reference standards. S4

Table S2. AFFF sample information and source details. S6

Table S3. Model configuration parameters for CFM-ID 4.0. ....S7

Table S4. Model configuration parameters for NPFAS-MS. S9

Table S5. PFAS candidates annotated across eight commercial AFFF products using the virtual library constructed with NPFAS-MS..S11

Table S6. PFAS candidates annotated in groundwater samples using the virtual library constructed with NPFAS-MS...S13

Figure S1. Workflow for MS/MS data acquisition and non-targeted analysis. S15

Figure S2. Representative spectra selection by MS-Clustering.......................................S16

Figure S3. Training and hold-out test set distribution of PFAS structures and spectra. S17

Figure S4. Tanimoto coefficient distribution between the training and hold-out test sets.S18

Figure S5. Transfer learning architecture of NPFAS-MS. S19

Figure S6. Representative examples illustrating the improved PFAS-specific fragmentation prediction of NPFAS-MS relative to the comparison models. S20

Figure S7. Representative cases illustrating two limitation patterns of NPFAS-MS spectral prediction. S21

Figure S8. Performance across five repeated random splits under four spectral similarity metrics (dot product, entropy similarity, dice coefficient, and precision). S22

Figure S9. UMAP visualization of chemical space coverage for training and hold-out test PFAS. S23

Figure S10. External validation of NPFAS-MS using two independent PFAS HRMS datasets. S24

Figure S11. Construction of a virtual PFAS mass spectral library...................................S25

Figure S12. PFAS annotation performance at different dot product score thresholds in environmental samples....S26

Figure S13. False positive rate of different dot product similarity thresholds using non-PFAS decoy dataset....S27

Figure S14. Byproduct PFAS detected in commercial AFFF products. S28

Figure S15. Structural annotation of (E)-5H-perfluorooct-6-ene-1-sulfonic acid (H-UPFOS) in AFFF products. S29

Figure S16. Representative emerging PFAS detected in groundwater samples. S30

Figure S17. Structural annotation of (E)-1,1,2,2,3,3,4,5,6,6,6-undecafluorohex-4-en-1-ol (UPFHxA)....S31

Figure S18. Validation of NPFAS-MS predicted spectra against reference standards across different environmental matrices. S32

References....S33

Table S1. PFAS reference standards.

Compound name	CAS No.	Supplier
1,1,2,2,3,3,4,4,4-nonafluoro-N,N-bis(2-hydroxyethyl)butane-1-sulfonamide (FBSEE diol)	34455-00-0	AK Scientific
N-[3-(dimethylamino)propyl]perfluoro-1-hexanesulfonamide (N-AP-FHxSA)	50598-28-2	Chiron AS
1H,1H,2H,2H-perfluorododecane sulfonic acid (10:2 FTS)	120226-60-0	Dr. Ehrenstorfer
2H-perfluoro-2-decenoic acid (FOUEA)	70887-84-2	Dr. Ehrenstorfer
2H-perfluoro-2-octenoic acid (FHUEA)	70887-88-6	Dr. Ehrenstorfer
1,1,2,2,3,3,4,4,4-nonafluoro-N-(2-hydroxyethyl)butane-1-sulfonamide (FBSE)	34454-99-4	Toronto Research Chemicals
bis[2-(perfluorooctyl)ethyl] phosphate (8:2 diPAP)	678-41-1	Toronto Research Chemicals
bis[2-(perfluorohexyl)ethyl] phosphate(6:2 diPAP)	57677-95-9	Toronto Research Chemicals
3,3,4,4,5,5,6,6,7,7,8,8,9,9,10,10,10-heptadecafluorodecanoic acid (8:2 FTCA)	27854-31-5	Toronto Research Chemicals
bis(perfluorohexyl)phosphinic acid (6:6 PFPi)	40143-77-9	Toronto Research Chemicals
perfluoro-n-butanoic acid (PFBA)	375-22-4	Wellington Laboratories
perfluoro-n-pentanoic acid (PFPeA)	2706-90-3	Wellington Laboratories
perfluorohexanoic acid (PFHxA)	307-24-4	Wellington Laboratories
perfluoroheptanoic acid (PFHpA)	375-85-9	Wellington Laboratories
perfluoro-n-octanoic acid (PFOA)	335-67-1	Wellington Laboratories
perfluorononanoic acid (PFNA)	375-95-1	Wellington Laboratories
perfluorodecanoic acid (PFDA)	335-76-2	Wellington Laboratories
perfluoroundecanoic acid (PFUnA)	2058-94-8	Wellington Laboratories
perfluorododecanoic acid (PFDoA)	307-55-1	Wellington Laboratories
perfluorotridecanoic acid (PFTrDA)	72629-94-8	Wellington Laboratories
perfluorotetradecanoic acid (PFTeDA)	376-06-7	Wellington Laboratories
perfluorobutane sulfonic acid (PFBS)	375-73-5	Wellington Laboratories
perfluoro-1-pentanesulfonic acid (PFPeS)	2706-91-4	Wellington Laboratories
perfluoro-1-hexane sulfonic acid (PFHxS)	355-46-4	Wellington Laboratories
perfluoro-1-heptane sulfonic acid (PFHpS)	375-92-8	Wellington Laboratories
perfluoro-1-octane sulfonic acid (PFOS)	1763-23-1	Wellington Laboratories
perfluoro-1-nonanesulfonate (PFNS)	68259-12-1	Wellington Laboratories
perfluoro-1-decane sulfonic acid (PFDS)	335-77-3	Wellington Laboratories
perfluoro-1-dodecanesulfonate (PFDoS)	79780-39-5	Wellington Laboratories
perfluoro-1-octanesulfonamide (PFOSA)	754-91-6	Wellington Laboratories
2-(N-ethylperfluoro-1-octanesulfonamido)-ethanol (NEtFOSE)	1691-99-2	Wellington Laboratories
2-(N-methylperfluoro-1-octanesulfonamido)-ethanol (NMeFOSE)	24448-09-7	Wellington Laboratories
N-ethylperfluoro-1-octanesulfonamide (NEtFOSA)	4151-50-2	Wellington Laboratories
N-methylperflouro-1-octanesulfonamide (NMeFOSA)	31506-32-8	Wellington Laboratories

N-methylperfluoro-1-octanesulfonamidoacetic acid (NMeFOSAA)	2355-31-9	Wellington Laboratories
N-ethylperfluoro-1-octanesulfonamidoacetic acid (NEtFOSAA)	2991-50-6	Wellington Laboratories
1H,1H,2H,2H-perfluorohexane sulfonic acid (4:2FTS)	757124-72-4	Wellington Laboratories
1H,1H,2H,2H-perfluorooctane sulfonic acid (6:2FTS)	27619-97-2	Wellington Laboratories
1H,1H,2H,2H-perfluorodecane sulfonic acid (8:2FTS)	39108-34-4	Wellington Laboratories
2-(heptafluoropropoxy)-2,3,3,3-tetrafluoropropionic acid (HFPO-DA)	13252-13-6	Wellington Laboratories
dodecafluoro-3H-4,8-dioxanonanoate (ADONA)	919005-14-4	Wellington Laboratories
perfluoro-4-oxapentanoic acid (PFMPA)	377-73-1	Wellington Laboratories
perfluoro-5-oxahexanoic acid (PFMBA)	863090-89-5	Wellington Laboratories
perfluoro-3,6-dioxaheptanoic acid (NFDHA)	151772-58-6	Wellington Laboratories
9-chlorohexadecafluoro-3-oxanonane-1-sulfonate (9Cl-PF3ONS)	756426-58-1	Wellington Laboratories
11-chloroeicosafluoro-3-oxaundecane-1-sulfonate (11Cl-PF3OUdS)	763051-92-9	Wellington Laboratories
perfluoro(2-ethoxyethane)sulfonate (PFEESA)	113507-82-7	Wellington Laboratories
2H,2H,3H,3H-perfluorohexanoic acid (3:3FTCA)	356-02-5	Wellington Laboratories
2H,2H,3H,3H-perfluorooctanoic acid (5:3FTCA)	914637-49-3	Wellington Laboratories
2H,2H,3H,3H-perfluorodecanoic acid (7:3FTCA)	812-70-4	Wellington Laboratories
2-(1,1,2,2,3,3,4,4,5,5,6,6,7,7,8,8,8-heptadecafluorooctylsulfonylamino)acetic acid (PFOSAA)	2806-24-8	Wellington Laboratories

Table S2. AFFF sample information and source details.

Sample ID	Sample type	Source	Supplier
AFFF-1	3% AFFF	Taiwan	Supplier-1
AFFF-2	3% AFFF	India	Supplier-2
AFFF-3	3% AFFF	Italy	Supplier-3
AFFF-4	3% AFFF	Unknown	Supplier-2
AFFF-5	3% AFFF	Unknown	Supplier-3
AFFF-6	3% AFFF	Australia	Supplier-4
AFFF-7	3% AFFF	China	Supplier-4
AFFF-8	3% AFFF	Unknown	Supplier-4

Table S3. Model configuration parameters for CFM-ID 4.0.

Category	Parameter	Index	Value
Basic	lambda	-	0
Basic	em_converge_thresh	-	0.01
Basic	ga_converge_thresh	-	0.001
Basic	model_depth	-	2
Basic	ionization_mode	-	2
Spectrum	spectrum_depth	0	2
Spectrum	spectrum_weight	0	1
Spectrum	spectrum_depth	1	2
Spectrum	spectrum_weight	1	1
Spectrum	spectrum_depth	2	2
Spectrum	spectrum_weight	2	1
Fragmentation	allow_frag_detours	-	1
Fragmentation	allow_intermediate_peak	-	1
Fragmentation	allow_cyclization	-	0
EM Algorithm	num_em_restarts	-	1
EM Algorithm	em_no_progress_count	-	2
EM Algorithm	em_max_iterations	-	100
GA Optimization	ga_method	-	2
GA Optimization	starting_step_size	-	0.001
GA Optimization	ending_step_size	-	0.00025
GA Optimization	ga_adam_beta_1	-	0.9
GA Optimization	ga_adam_beta_2	-	0.999
GA Optimization	ga_max_iterations	-	30
GA Optimization	ga_sampling_method	-	4
GA Optimization	ga_minibatch_nth_size	-	20
Post-Processing	default_predicted_peak_min	-	1
Post-Processing	default_predicted_peak_max	-	30
Post-Processing	default_predicted_min_intensity	-	0
Post-Processing	default_postprocessing_energy	-	80
Neural Network	theta_function	-	2

Neural Network	theta_nn_hlayer_num_nodes	0	128
Neural Network	theta_nn_hlayer_num_nodes	1	128
Neural Network	theta_nn_layer_act_func_ids	0	2
Neural Network	theta_nn_layer_act_func_ids	1	2
Neural Network	theta_nn_layer_act_func_ids	2	0
Neural Network	nn_layer_dropout_probs	0	0.1
Neural Network	nn_layer_dropout_probs	1	0.1
Neural Network	nn_layer_dropout_probs	2	0
Technical	collected_all_used_idx	-	1

Table S4. Model configuration parameters for NPFAS-MS.

Category	Parameter	Index	Value
Basic	lambda	-	0
Basic	em_converge_thresh	-	0.005
Basic	ga_converge_thresh	-	0.001
Basic	model_depth	-	2
Basic	ionization_mode	-	2
Spectrum	spectrum_depth	0	2
Spectrum	spectrum_weight	0	1
Spectrum	spectrum_depth	1	2
Spectrum	spectrum_weight	1	1
Spectrum	spectrum_depth	2	2
Spectrum	spectrum_weight	2	1
Fragmentation	allow_frag_detours	-	1
Fragmentation	allow_intermediate_peak	-	1
Fragmentation	allow_cyclization	-	0
EM Algorithm	num_em_restarts	-	1
EM Algorithm	em_no_progress_count	-	2
EM Algorithm	em_max_iterations	-	20
GA Optimization	ga_method	-	2
GA Optimization	starting_step_size	-	0.001
GA Optimization	ending_step_size	-	0.00025
GA Optimization	ga_adam_beta_1	-	0.9
GA Optimization	ga_adam_beta_2	-	0.999
GA Optimization	ga_max_iterations	-	30
GA Optimization	ga_sampling_method	-	4
GA Optimization	ga_minibatch_nth_size	-	2
Post-Processing	default_predicted_peak_min	-	1
Post-Processing	default_predicted_peak_max	-	10000
Post-Processing	default_predicted_min_intensity	-	3
Post-Processing	default_postprocessing_energy	-	100
Neural Network	theta_function	-	2

Neural Network	theta_nn_hlayer_num_nodes	0	128
Neural Network	theta_nn_hlayer_num_nodes	1	128
Neural Network	theta_nn_layer_act_func_ids	0	2
Neural Network	theta_nn_layer_act_func_ids	1	2
Neural Network	theta_nn_layer_act_func_ids	2	0
Neural Network	nn_layer_dropout_probs	0	0.1
Neural Network	nn_layer_dropout_probs	1	0.1
Neural Network	nn_layer_dropout_probs	2	0
Neural Network	nn_layer_freeze	0	1
Neural Network	nn_layer_freeze	1	1
Neural Network	nn_layer_freeze	2	0
Technical	collected_all_used_idx	-	1

Table S5. PFAS candidates annotated across eight commercial AFFF products using the virtual library constructed with NPFAS-MS.

Compound name	Molecular formula	SMILES
2,2,2-Trifluoroethyl dihydrogen phosphate	$C_2H_4F_3O_4P$	OP(O)(=O)OCC(F)(F)F
2-[(1,1,1,3,3,3-Hexafluoropropan-2-yl)oxy]ethan-1-ol	$C_5H_6F_6O_2$	OCCOC(C(F)(F)F)C(F)(F)F
Heptafluorobutanoic acid	$C_4HF_7O_2$	OC(=O)C(F)(F)C(F)(F)C(F)(F)F
Ethyl 2,2,3,4,4,4-hexafluorobutanoate	$C_6H_6F_6O_2$	CCOC(=O)C(F)(F)C(F)C(F)(F)F
2,2,3,3-Tetrafluoro-3-sulfopropanoic acid	$C_3H_2F_4O_5S$	OC(=O)C(F)(F)C(F)(F)S(O)(=O)=O
2,2,3,3,5,5-Hexafluoro-4-(trifluoromethyl)oxolane	C_5HF_9O	FC(F)(F)C1C(F)(F)OC(F)(F)C1(F)F
Nonafluoropentanoic acid	$C_5HF_9O_2$	OC(=O)C(F)(F)C(F)(F)C(F)(F)C(F)(F)F
1,1,1,2,2,3,3,4,5,5,5-Undecafluoropentane	C_5HF_{11}	FC(C(F)(F)F)C(F)(F)C(F)(F)C(F)(F)F
1,1,2,3,3,3-Hexafluoro-2-(trifluoromethyl)propane-1-sulfonic acid	$C_4HF_9O_3S$	OS(=O)(=O)C(F)(F)C(F)(C(F)(F)F)C(F)(F)F
Nonafluorobutane-1-sulfonic acid	$C_4HF_9O_3S$	OS(=O)(=O)C(F)(F)C(F)(F)C(F)(F)C(F)(F)F
2,2,3,4,4,5,5,5-Octafluoro-3-(trifluoromethyl)pentanoic acid	$C_6HF_{11}O_2$	OC(=O)C(F)(F)C(F)(C(F)(F)F)C(F)(F)C(F)(F)F
Undecafluorohexanoic acid	$C_6HF_{11}O_2$	OC(=O)C(F)(F)C(F)(F)C(F)(F)C(F)(F)C(F)(F)F
1,1,2,2-Tetrafluoro-2-(pentafluoroethoxy)ethane-1-sulfonic acid	$C_4HF_9O_4S$	OS(=O)(=O)C(F)(F)C(F)(F)OC(F)(F)C(F)(F)F
1,1,1,2,2,3,3,4,5,5,6,6,6-Tridecafluorohexane	C_6HF_{13}	FC(C(F)(F)C(F)(F)F)C(F)(F)C(F)(F)C(F)(F)F
Undecafluoropentane-1-sulfonic acid	$C_5HF_{11}O_3S$	OS(=O)(=O)C(F)(F)C(F)(F)C(F)(F)C(F)(F)C(F)(F)F
Tridecafluoroheptanoic acid	$C_7HF_{13}O_2$	OC(=O)C(F)(F)C(F)(F)C(F)(F)C(F)(F)C(F)(F)C(F)(F)F
1,1,2,2,3,3,4,4,5,5,6,6,6-Tridecafluorohexane-1-sulfonamide	$C_6H_2F_{13}NO_2S$	NS(=O)(=O)C(F)(F)C(F)(F)C(F)(F)C(F)(F)C(F)(F)C(F)(F)F
Tridecafluorohexane-1-sulfonic acid	$C_6HF_{13}O_3S$	OS(=O)(=O)C(F)(F)C(F)(F)C(F)(F)C(F)(F)C(F)(F)C(F)(F)F
2,3,3,4,4,5,6,6,6-Nonafluoro-2,5-bis(trifluoromethyl)hexanoic acid	$C_8HF_{15}O_2$	OC(=O)C(F)(C(F)(F)F)C(F)(F)C(F)(F)C(F)(C(F)(F)F)C(F)(F)F
Pentadecafluorooctanoic acid	$C_8HF_{15}O_2$	OC(=O)C(F)(F)C(F)(F)C(F)(F)C(F)(F)C(F)(F)C(F)(F)C(F)(F)F
3,3,4,4,5,5,6,6,7,7,8,8,8-Tridecafluorooctane-1-sulfonic acid	$C_8H_5F_{13}O_3S$	OS(=O)(=O)CCC(F)(F)C(F)(F)C(F)(F)C(F)(F)C(F)(F)C(F)(F)F
(E)-5H-Perfluorooct-6-ene-1-sulfonic acid	$C_8H_2F_{14}O_3S$	OS(=O)(=O)C(F)(F)C(F)(F)C(F)(F)C(F)(F)C(F)C(\F)=C(/F)C(F)(F)F

Pentadecafluoroheptane-1-sulfonic acid	$C_7HF_{15}O_3S$	OS(=O)(=O)C(F)(F)C(F)(F)C(F)(F)C(F)(F)C(F)(F)C(F)(F)C(F)(F)F
Perfluoro-3-propylcyclopentane-1-sulfonic acid	$C_8HF_{15}O_3S$	OS(=O)(=O)C1(F)C(F)(F)C(F)(F)C(F)(C(F)(F)C(F)(F)C(F)(F)F)C1(F)F
7H-Perfluorooctanesulfonic acid	$C_8H_2F_{16}O_3S$	OS(=O)(=O)C(F)(F)C(F)(F)C(F)(F)C(F)(F)C(F)(F)C(F)(F)C(F)C(F)(F)F
1,1,2,2,3,3,4,4,5,5,6,6,7,7,8,8-Hexadecafluorooctane-1-sulfonic acid	$C_8H_2F_{16}O_3S$	[H]C(F)(F)C(F)(F)C(F)(F)C(F)(F)C(F)(F)C(F)(F)C(F)(F)C(F)(F)S(O)(=O)=O
N-[3-(Dimethylamino)propyl]-1,1,2,2,3,3,4,4,5,5,6,6,6-tridecafluorohexane-1-sulfonamide	$C_{11}H_{13}F_{13}N_2O_2S$	CN(C)CCCNS(=O)(=O)C(F)(F)C(F)(F)C(F)(F)C(F)(F)C(F)(F)C(F)(F)F
1,1,2,2,3,3,4,4,5,5,6,6,7,7,8,8,8-Heptadecafluorooctane-1-sulfonamide	$C_8H_2F_{17}NO_2S$	NS(=O)(=O)C(F)(F)C(F)(F)C(F)(F)C(F)(F)C(F)(F)C(F)(F)C(F)(F)C(F)(F)F
Heptadecafluorooctane-2-sulfonic acid	$C_8HF_{17}O_3S$	OS(=O)(=O)C(F)(C(F)(F)F)C(F)(F)C(F)(F)C(F)(F)C(F)(F)C(F)(F)C(F)(F)F
1,1,2,2,3,3,4,4,5,5,6,7,7,7-Tetradecafluoro-6-(trifluoromethyl)heptane-1-sulfonic acid	$C_8HF_{17}O_3S$	OS(=O)(=O)C(F)(F)C(F)(F)C(F)(F)C(F)(F)C(F)(F)C(F)(C(F)(F)F)C(F)(F)F
1,1,2,2,3,4,4,5,5,6,6,7,7,7-Tetradecafluoro-3-(trifluoromethyl)heptane-1-sulfonic acid	$C_8HF_{17}O_3S$	OS(=O)(=O)C(F)(F)C(F)(F)C(F)(C(F)(F)F)C(F)(F)C(F)(F)C(F)(F)C(F)(F)F
Heptadecafluorooctane-1-sulfonic acid	$C_8HF_{17}O_3S$	OS(=O)(=O)C(F)(F)C(F)(F)C(F)(F)C(F)(F)C(F)(F)C(F)(F)C(F)(F)C(F)(F)F
3,3,4,4,5,5,6,6,7,7,8,8,9,9,10,10,10-Heptadecafluorodecane-1-sulfonic acid	$C_{10}H_5F_{17}O_3S$	OS(=O)(=O)CCC(F)(F)C(F)(F)C(F)(F)C(F)(F)C(F)(F)C(F)(F)C(F)(F)C(F)(F)F
N-[3-(Dimethylamino)propyl]-1,1,2,2,3,3,4,4,5,5,6,6,7,7,7-pentadecafluoroheptane-1-sulfonamide	$C_{12}H_{13}F_{15}N_2O_2S$	CN(C)CCCNS(=O)(=O)C(F)(F)C(F)(F)C(F)(F)C(F)(F)C(F)(F)C(F)(F)C(F)(F)F
Nonadecafluorononane-1-sulfonic acid	$C_9HF_{19}O_3S$	OS(=O)(=O)C(F)(F)C(F)(F)C(F)(F)C(F)(F)C(F)(F)C(F)(F)C(F)(F)C(F)(F)C(F)(F)F
N-ethyl-1,1,2,2,3,3,4,4,5,5,6,6,6-tridecafluoro-N-[2-[2-(2-hydroxyethoxy)ethoxy]ethyl]hexane-1-sulfonamide	$C_{14}H_{18}F_{13}NO_5S$	OCCOCCOCCN(CC)S(=O)(=O)C(F)(F)C(F)(F)C(F)(F)C(F)(F)C(F)(F)C(F)(F)F

Table S6. PFAS candidates annotated in groundwater samples using the virtual library constructed with NPFAS-MS.

**Compound name**	**Molecular formula**	**SMILES**
Trifluoromethanesulfonic acid	CHF_3O_3S	OS(=O)(=O)C(F)(F)F
Pentafluoropropanoic acid	$C_3HF_5O_2$	OC(=O)C(F)(F)C(F)(F)F
Difluoromethyl trifluoroacetate	$C_3HF_5O_2$	FC(F)OC(=O)C(F)(F)F
2,3,6-Trifluorobenzene-1-thiol	$C_6H_3F_3S$	FC1=C(F)C(S)=C(F)C=C1
1,1,1,2,3,3,3-Heptafluoropropane	C_3HF_7	FC(C(F)(F)F)C(F)(F)F
1,2,2,2-Tetrafluoroethane-1-sulfonyl fluoride	$C_2HF_5O_2S$	FC(C(F)(F)F)S(F)(=O)=O
(E)-1,1,2,3,4,4,4-heptafluorobut-2-en-1-ol	C_4HF_7O	OC(F)(F)/C(F)=C(C(F)(F)F)F
1,1,1,2,2,3,3,4,4-Nonafluorobutane	C_4HF_9	FC(F)C(F)(F)C(F)(F)C(F)(F)F
2,2,3,3,5,5-Hexafluoro-4-(trifluoromethyl)oxolane	C_5HF_9O	FC(F)(F)C1C(F)(F)OC(F)(F)C1(F)F
Heptafluoropropane-1-sulfonic acid	$C_3HF_7O_3S$	OS(=O)(=O)C(F)(F)C(F)(F)C(F)(F)F
E)-1,1,2,2,3,3,4,5,6,6,6-undecafluorohex-4-en-1-ol	$C_6HF_{11}O$	OC(F)(F)C(F)(F)C(F)(F)/C(F)=C(C(F)(F)F)F
1,1,2,2,3,3,4,4,4-Nonafluorobutane-1-sulfonamide	$C_4H_2F_9NO_2S$	[H]N([H])S(=O)(=O)C(F)(F)C(F)(F)C(F)(F)C(F)(F)F
1,1,2,3,3,3-Hexafluoro-2-(trifluoromethyl)propane-1-sulfonic acid	$C_4HF_9O_3S$	OS(=O)(=O)C(F)(F)C(F)(C(F)(F)F)C(F)(F)F
1,1,2,2-Tetrafluoro-2-(1,2,2,2-tetrafluoroethoxy)ethane-1-sulfonyl fluoride	$C_4HF_9O_3S$	FC(OC(F)(F)C(F)(F)S(F)(=O)=O)C(F)(F)F
Nonafluorobutane-1-sulfonic acid	$C_4HF_9O_3S$	OS(=O)(=O)C(F)(F)C(F)(F)C(F)(F)C(F)(F)F
6,7-Bis(trifluoromethyl)-1,3-benzothiazole-2(3H)-thione	$C_9H_3F_6NS_2$	FC(F)(F)C1=C(C2=C(NC(=S)S2)C=C1)C(F)(F)F
1,1,1,2,2,3,3,4,4,5,6,6,6-Tridecafluorohexane	C_6HF_{13}	FC(C(F)(F)F)C(F)(F)C(F)(F)C(F)(F)C(F)(F)F
(E)-1,1,2,2,3,3,4,4,5,6,7,7,7-tridecafluorohept-5-en-1-ol	$C_7HF_{13}O$	OC(F)(F)C(F)(F)C(F)(F)C(F)(F)/C(F)=C(C(F)(F)F)F
1,1,1,2,2,3,3-Heptafluoro-4,4-bis(trifluoromethyl)hexane	$C_8H_5F_{13}$	CCC(C(F)(F)F)(C(F)(F)F)C(F)(F)C(F)(F)C(F)(F)F
Undecafluoropentane-1-sulfonic acid	$C_5HF_{11}O_3S$	OS(=O)(=O)C(F)(F)C(F)(F)C(F)(F)C(F)(F)C(F)(F)F
(Perfluorobutyl) sulfonamido acetic acid	$C_6H_4F_9NO_4S$	OC(=O)CNS(=O)(=O)C(F)(F)C(F)(F)C(F)(F)C(F)(F)F
1,1,1,2,2,3,3,4,4,5,5,6,6,7,7-Pentadecafluoroheptane	C_7HF_{15}	FC(F)C(F)(F)C(F)(F)C(F)(F)C(F)(F)C(F)(F)C(F)(F)F
1,1,2,2,3,3,4,4,5,5,6,6,6-Tridecafluorohexane-1-sulfonamide	$C_6H_2F_{13}NO_2S$	NS(=O)(=O)C(F)(F)C(F)(F)C(F)(F)C(F)(F)C(F)(F)C(F)(F)F
Tridecafluorohexane-1-sulfonic acid	$C_6HF_{13}O_3S$	OS(=O)(=O)C(F)(F)C(F)(F)C(F)(F)C(F)(F)C(F)(F)C(F)(F)F
4-Chlorophenyl (2,2,3,3,4,4,4-heptafluorobutanoyl)sulfamate	$C_{10}H_5ClF_7NO_4S$	FC(F)(F)C(F)(F)C(F)(F)C(=O)NS(=O)(=O)OC1=CC=C(Cl)C=C1

3,3,4,4,5,5,6,6,7,7,8,8,8-Tridecafluorooctane-1-sulfonic acid	$C_8H_5F_{13}O_3S$	OS(=O)(=O)CCC(F)(F)C(F)(F)C(F)(F)C(F)(F)C(F)(F)C(F)(F)F
Pentadecafluoroheptane-1-sulfonic acid	$C_7HF_{15}O_3S$	OS(=O)(=O)C(F)(F)C(F)(F)C(F)(F)C(F)(F)C(F)(F)C(F)(F)C(F)(F)F
Perfluoro-4-ethylcyclohexane	$C_8HF_{15}O_3S$	C1(C(C(C(C(C1(F)F)(F)F)(F)S(=O)(=O)O)(F)F)(F)F)(C(C(F)(F)F)(F)F)F
Perfluoro-3-propylcyclopentane-1-sulfonic acid	$C_8HF_{15}O_3S$	OS(=O)(=O)C1(F)C(F)(F)C(F)(F)C(F)(C(F)(F)C(F)(F)C(F)(F)F)C1(F)F
2,2,3,3,4,4,4-Heptafluorobutyl nonafluorobutane-1-sulfonate	$C_8H_2F_{16}O_3S$	FC(F)(F)C(F)(F)C(F)(F)COS(=O)(=O)C(F)(F)C(F)(F)C(F)(F)C(F)(F)F
1,1,2,2,3,3,4,4,5,5,6,6,7,7,8,8-Hexadecafluorooctane-1-sulfonic acid	$C_8H_2F_{16}O_3S$	[H]C(F)(F)C(F)(F)C(F)(F)C(F)(F)C(F)(F)C(F)(F)C(F)(F)C(F)(F)S(O)(=O)=O
1,1,2,2,3,3,4,4,5,5,6,6,7,7,8,8,8-Heptadecafluorooctane-1-sulfonamide	$C_8H_2F_{17}NO_2S$	NS(=O)(=O)C(F)(F)C(F)(F)C(F)(F)C(F)(F)C(F)(F)C(F)(F)C(F)(F)C(F)(F)F
Heptadecafluorooctane-2-sulfonic acid	$C_8HF_{17}O_3S$	OS(=O)(=O)C(F)(C(F)(F)F)C(F)(F)C(F)(F)C(F)(F)C(F)(F)C(F)(F)C(F)(F)F
1,1,2,2,3,3,4,4,5,5,6,6,7,7,7-Tetradecafluoro-3-(trifluoromethyl)heptane-1-sulfonic acid	$C_8HF_{17}O_3S$	OS(=O)(=O)C(F)(F)C(F)(F)C(F)(C(F)(F)F)C(F)(F)C(F)(F)C(F)(F)C(F)(F)F
1,1,2,2,3,3,4,4,5,5,6,7,7,7-Tetradecafluoro-6-(trifluoromethyl)heptane-1-sulfonic acid	$C_8HF_{17}O_3S$	OS(=O)(=O)C(F)(F)C(F)(F)C(F)(F)C(F)(F)C(F)(F)C(F)(C(F)(F)F)C(F)(F)F
Heptadecafluorooctane-1-sulfonic acid	$C_8HF_{17}O_3S$	OS(=O)(=O)C(F)(F)C(F)(F)C(F)(F)C(F)(F)C(F)(F)C(F)(F)C(F)(F)C(F)(F)F
1,1,2,2,3,3,4,5,5,6,6,7,7,7-Tetradecafluoro-4-(trifluoromethyl)heptane-1-sulfonic acid	$C_8HF_{17}O_3S$	OS(=O)(=O)C(F)(F)C(F)(F)C(F)(F)C(F)(C(F)(F)F)C(F)(F)C(F)(F)C(F)(F)F
Perfluorooctyl hydrogen sulfate	$C_8HF_{17}O_4S$	OS(=O)(=O)OC(F)(F)C(F)(F)C(F)(F)C(F)(F)C(F)(F)C(F)(F)C(F)(F)C(F)(F)F
Nonadecafluorononane-1-sulfonic acid	$C_9HF_{19}O_3S$	OS(=O)(=O)C(F)(F)C(F)(F)C(F)(F)C(F)(F)C(F)(F)C(F)(F)C(F)(F)C(F)(F)C(F)(F)F
1,1,2,2,3,3,4,4,4-Nonafluoro-N-(1,1,2,2,3,3,4,4,4-nonafluorobutane-1-sulfonyl)butane-1-sulfonamide	$C_8HF_{18}NO_4S_2$	[H]N(S(=O)(=O)C(F)(F)C(F)(F)C(F)(F)C(F)(F)F)S(=O)(=O)C(F)(F)C(F)(F)C(F)(F)C(F)(F)F

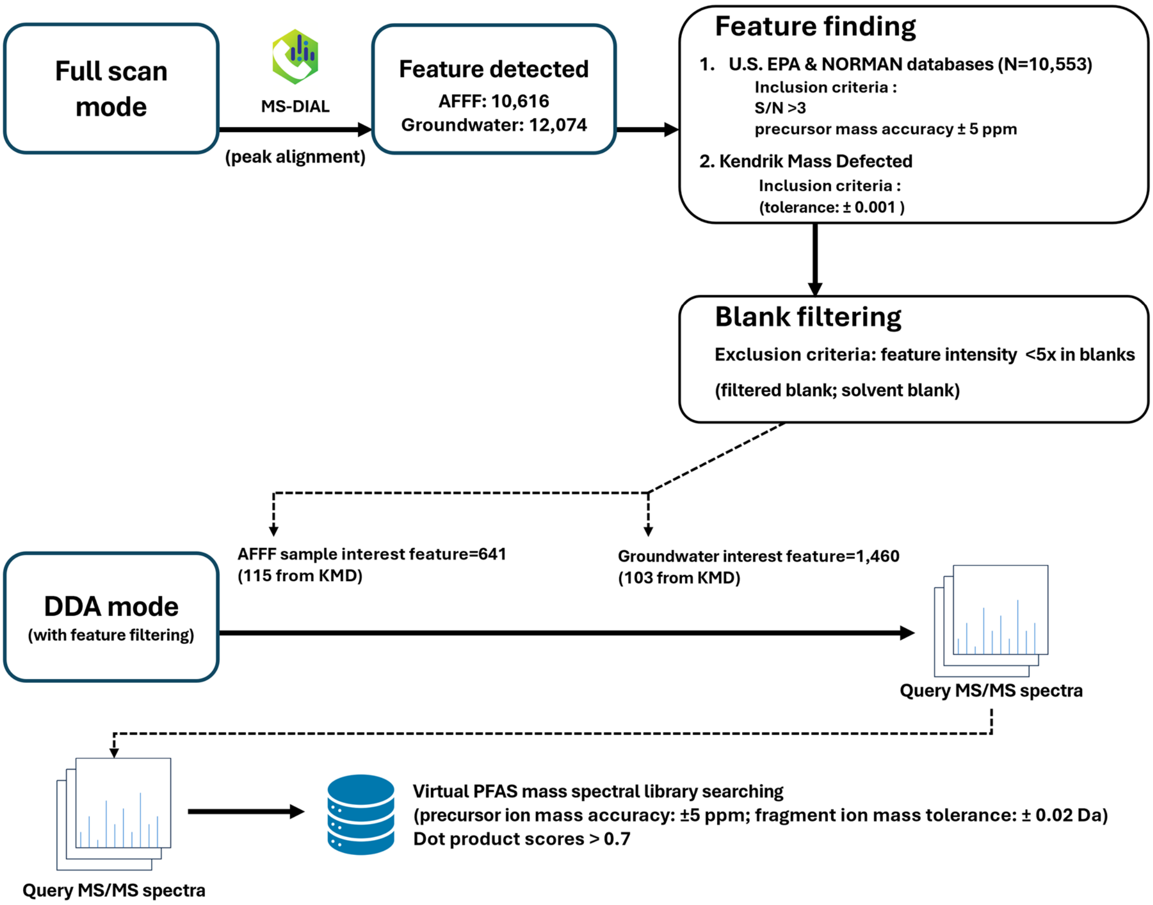

Figure S1. Workflow for MS/MS data acquisition and non-targeted analysis. The MS/MS data acquisition consists of two main stages: (1) Interest feature finding: MS-DIAL software was used for peak alignment of full scan MS data, followed by feature finding against U.S. EPA and NORMAN databases (n=10,553) with inclusion criteria of S/N > 3 and precursor mass accuracy ±5 ppm, and Kendrick Mass Defect (KMD) with an inclusion tolerance of ±0.001. Features were then subjected to blank samples filtering with an exclusion criterion of feature intensity < 5× in blanks (filtered blank, solvent blank). (2) Data-dependent acquisition (DDA) mode with interest features: After feature prioritization, groundwater sample and AFFF sample were reinjected for MS/MS spectrum acquisition in DDA mode. The acquired MS/MS spectra were then matched against the virtual PFAS mass spectral library using precursor ion mass accuracy of ±5 ppm, fragment ion mass tolerance of ±0.02 Da, with a dot product score >0.7 for structure annotation.

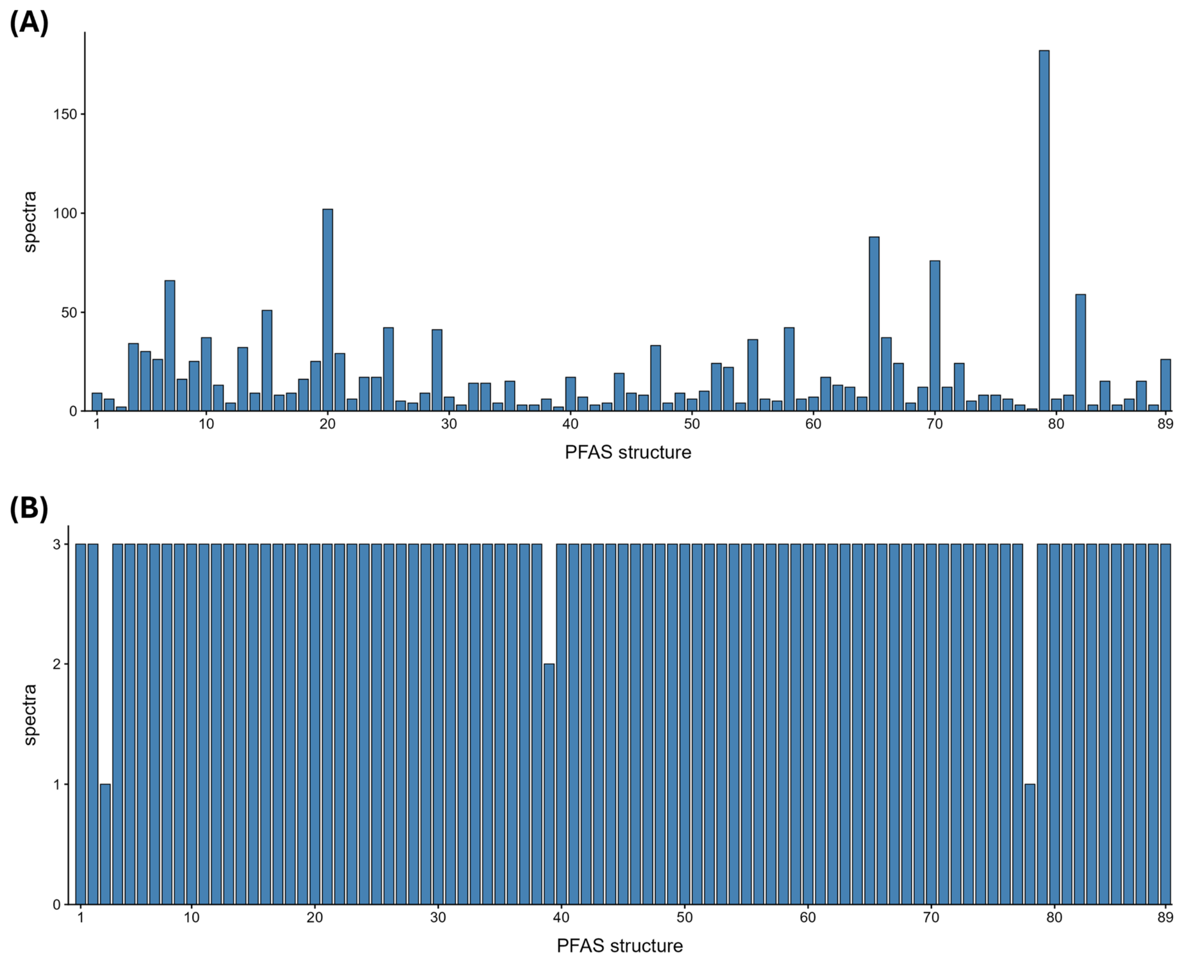

Figure S2. Representative spectra selection by MS-Clustering.[1] (A) Distribution of spectra count per PFAS structure before MS-Clustering processing, where the x-axis represents the 89 PFAS collected from public mass spectral databases (1–89) and the y-axis shows their spectra count, revealing severe data imbalance with some PFAS having numerous spectra. (B) Distribution of spectrum count per PFAS structure after MS-Clustering processing.

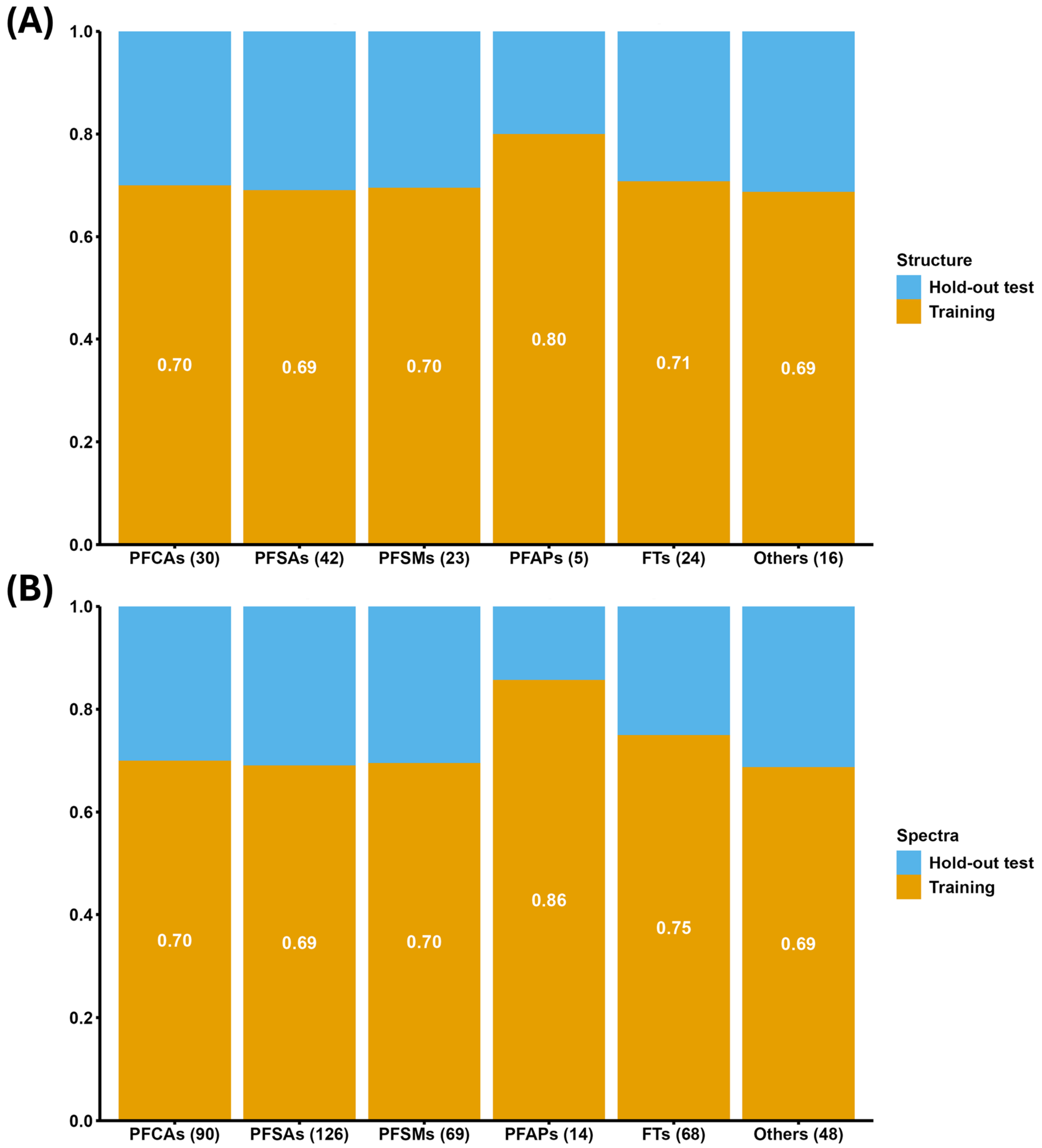

Figure S3. Training and hold-out test set distribution of PFAS structures and spectra. Distribution of (A) 140 PFAS structures and (B) 415 MS/MS spectra across six different categories. Data were split with an approximate 70:30 ratio into training (orange) and hold-out test (blue) sets. Numbers in parentheses indicate total counts per category, and labeled values represent training set proportions.

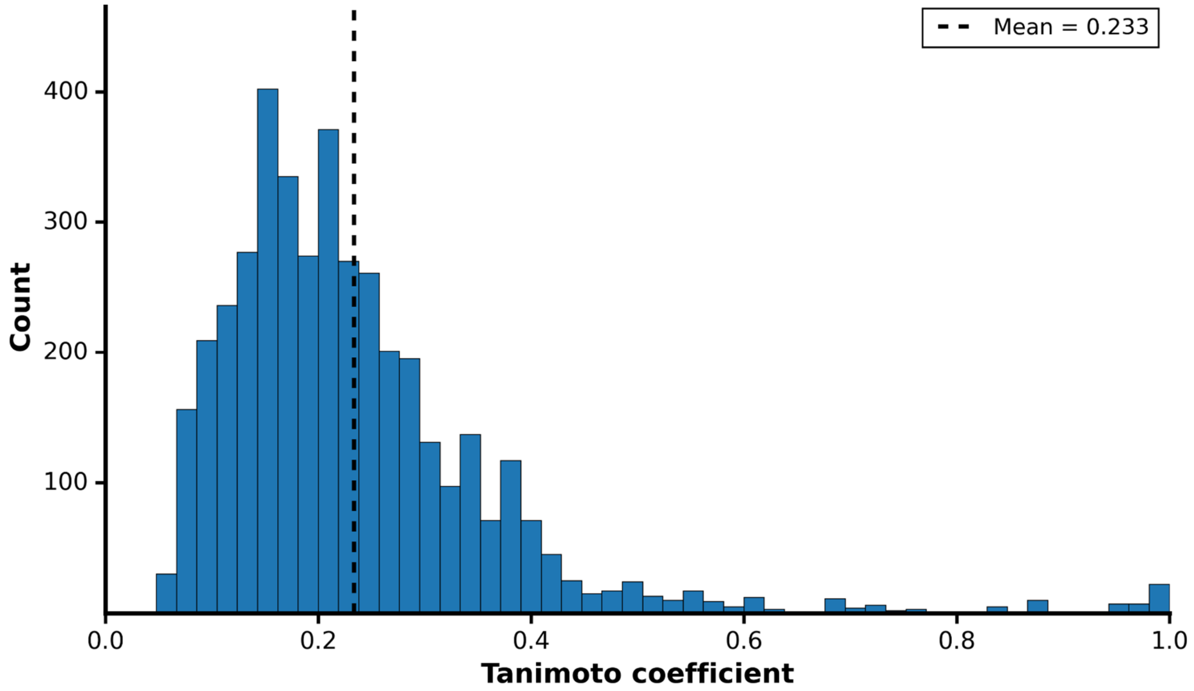

Figure S4. Tanimoto coefficient distribution between the training and hold-out test sets. For each PFAS in the hold-out test set, the Tanimoto coefficient against all training set PFAS was calculated using ECFP_6 fingerprints. The dashed line indicates the mean of Tanimoto coefficient (0.233).

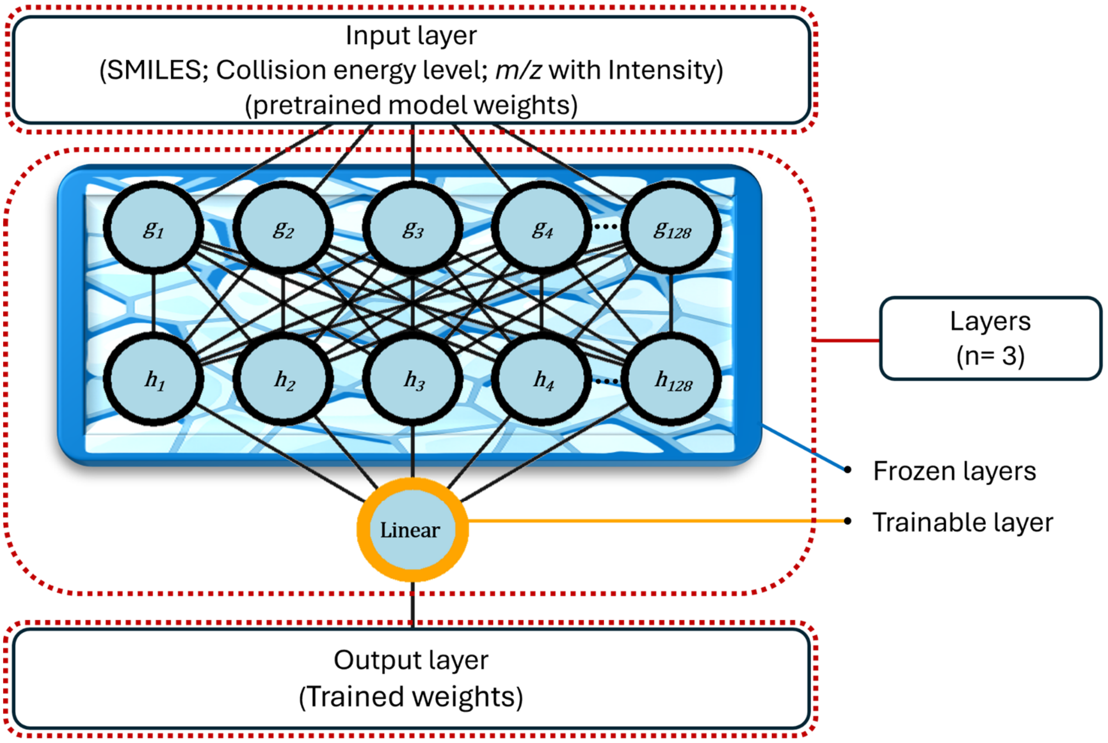

Figure S5. Transfer learning architecture of NPFAS-MS. The pretrained CFM-ID 4.0 model[2] was fine-tuned for PFAS MS/MS spectrum prediction by freezing all neural network layers while keeping only the final linear layer trainable (orange).[3] The input layer receives molecular structure information as SMILES, collision energy level, and *m/z* with intensity values. The output layer contains the fine-tuned weights for PFAS-specific spectra prediction.

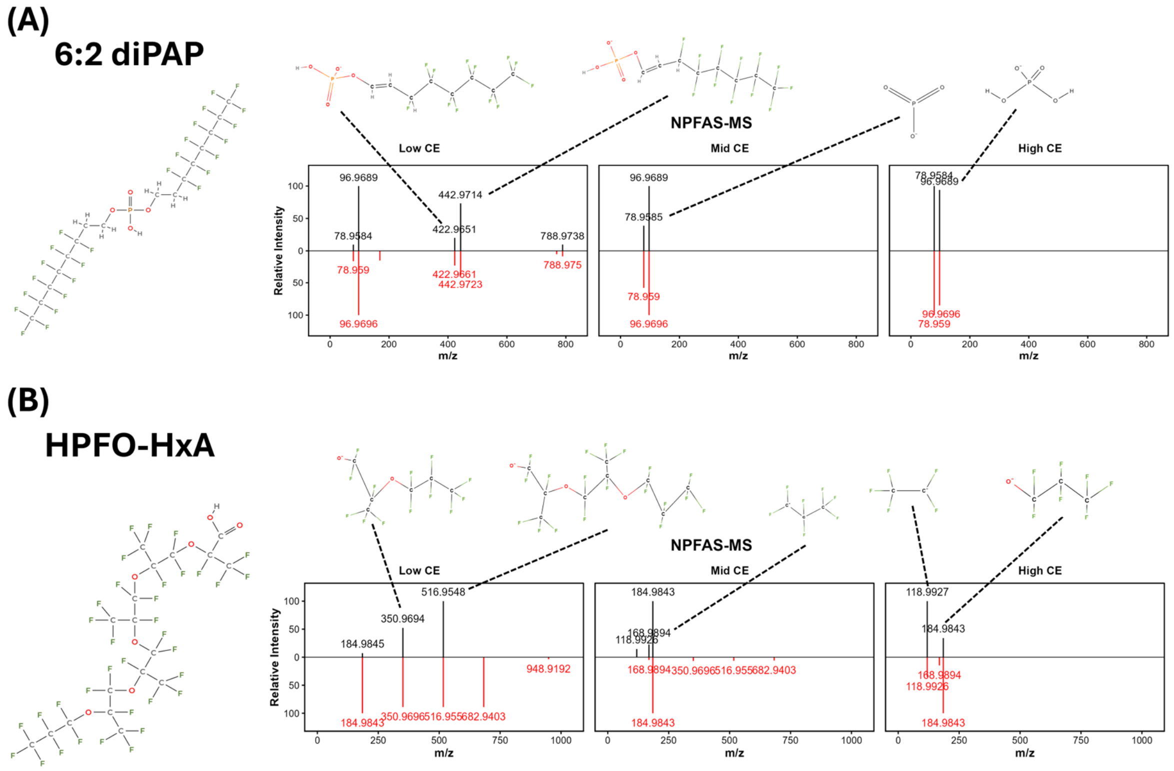

Figure S6. Representative examples illustrating the improved PFAS-specific fragmentation prediction of NPFAS-MS relative to the comparison models. (A) 6:2 diPAP acquired from reference standard analysis, NPFAS-MS successfully reproduced the major experimentally observed fragment ions across all collision energy (CE) levels, including the characteristic phosphate head group ions at *m/z* 78.9590 (PO_3^-) and 96.9696 ($H_2PO_4^-$), as well as the phosphate monoester ions at *m/z* 422.9661 and 442.9723. (B) HPFO-HxA obtained from public mass spectral databases, NPFAS-MS successfully predicted the major perfluoroalkyl ether chain fragment series at *m/z* 184.9843, 350.9696, and 516.9550 at low CE, as well as *m/z* 168.9894 at medium CE level and both *m/z* 118.9926 ($C_2F_5^-$) and 168.9894 ($C_3F_7^-$) at high CE level. These results show that NPFAS-MS not only reproduces diagnostic ions but also captures fluorinated backbone-derived fragments that are highly informative for PFAS subclass differentiation, thereby contributing to improved spectral matching precision.

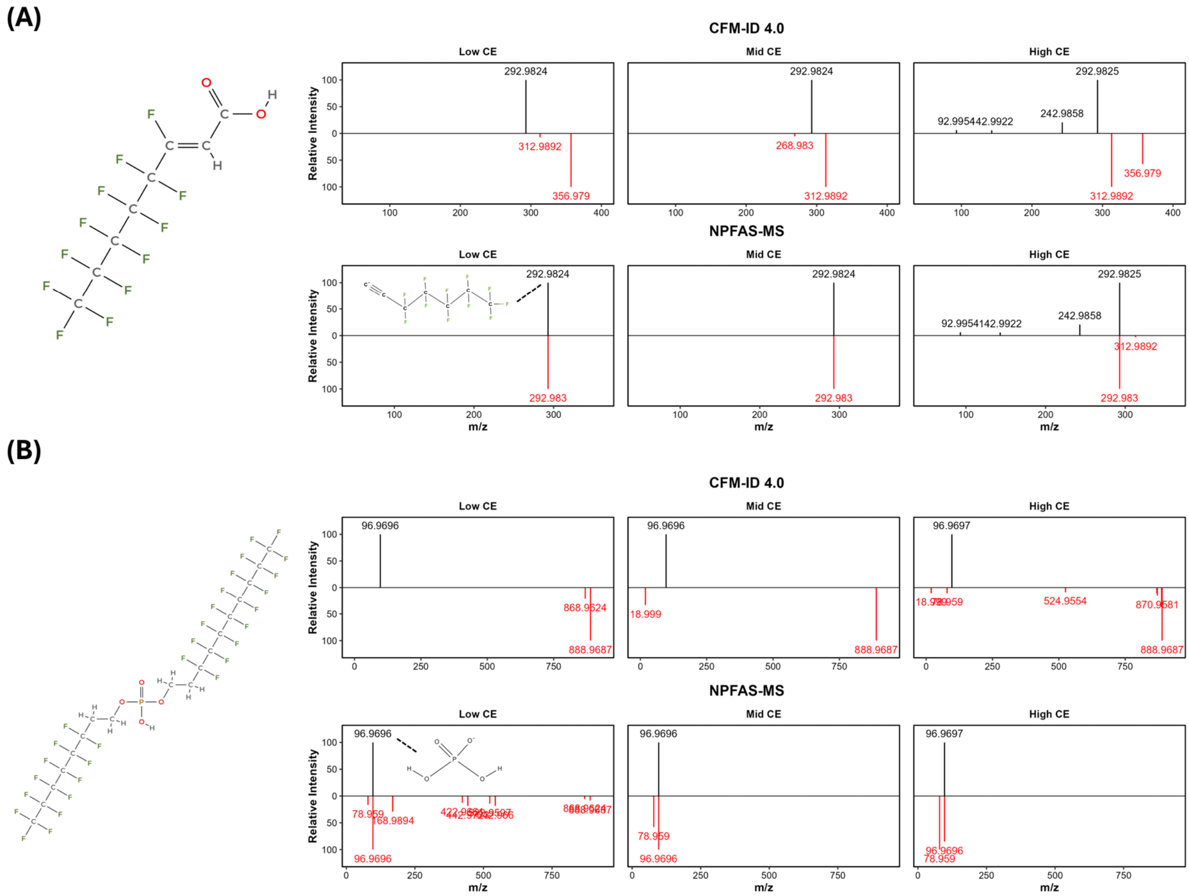

Figure S7. Representative cases illustrating two limitation patterns of NPFAS-MS spectral prediction. (A) Comparison of experimental (black, upper panels) and predicted (red, lower panels) MS/MS spectra of 2H-perfluoro-2-octenoic acid (FHUEA) at low, mid, and high CE levels for CFM-ID 4.0 and NPFAS-MS. NPFAS-MS successfully predicted the major product ion at *m/z* 292.983 ($C_7F_{11}^-$), a structurally diagnostic fragment rather than the precursor ion, but failed to reproduce additional lower-intensity fragment ions at high CE level. (B) Comparison of experimental and predicted MS/MS spectra of (perfluorohexyl)ethyl (perfluorooctyl)ethyl hydrogen phosphate (6:2/8:2 diPAP) at low, mid, and high CE levels. NPFAS-MS successfully predicted the characteristic product ion at *m/z* 96.9696 ($H_2PO_4^-$), which CFM-ID 4.0 failed to predict, but over-predicted additional low-intensity ions at low CE level, reducing the dot-product similarity score.

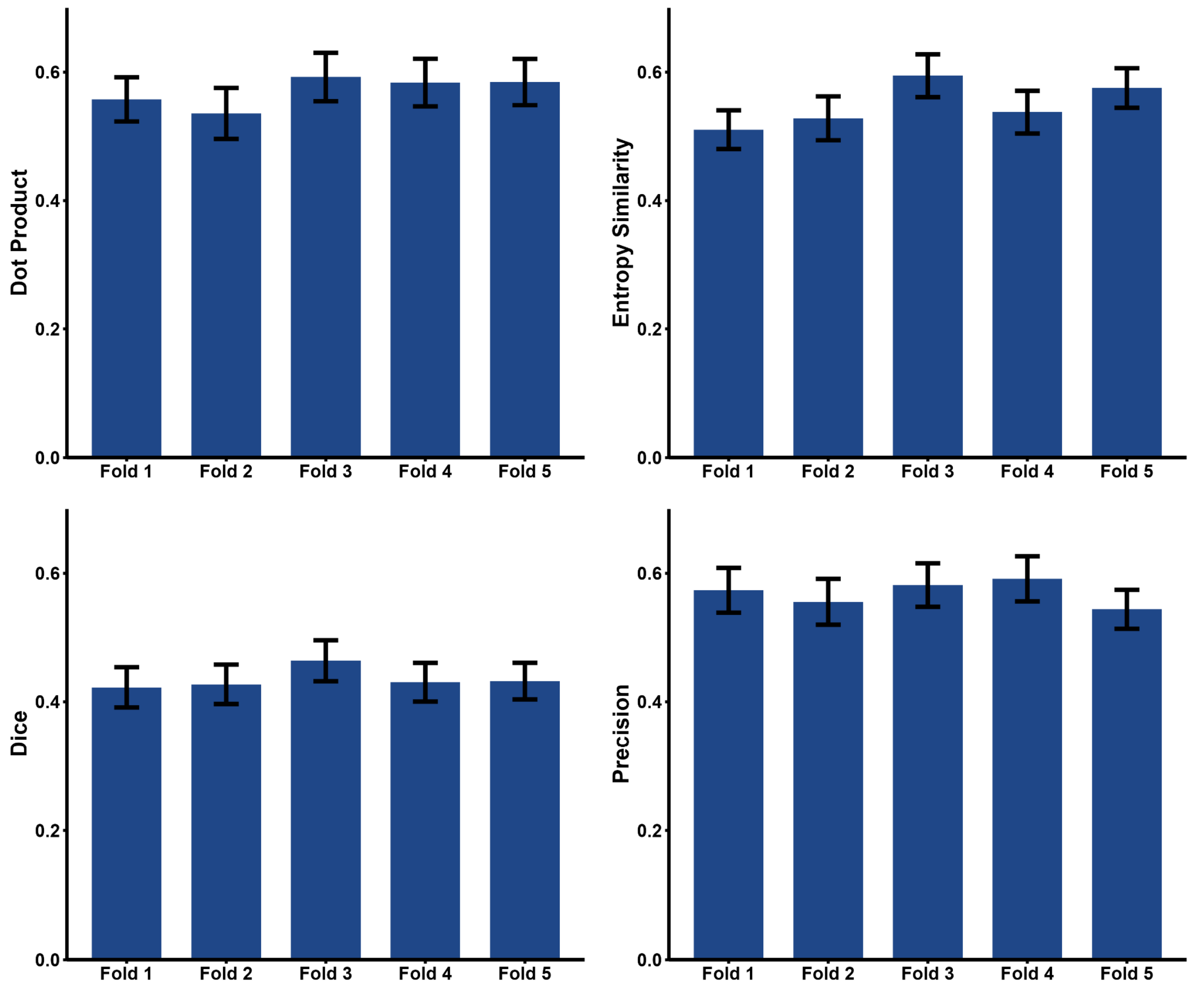

Figure S8. Performance across five repeated random splits under four spectral similarity metrics (dot product, entropy similarity, dice coefficient, and precision). No statistically significant differences were observed across splits by one-way ANOVA ($p > 0.05$ for all metrics).

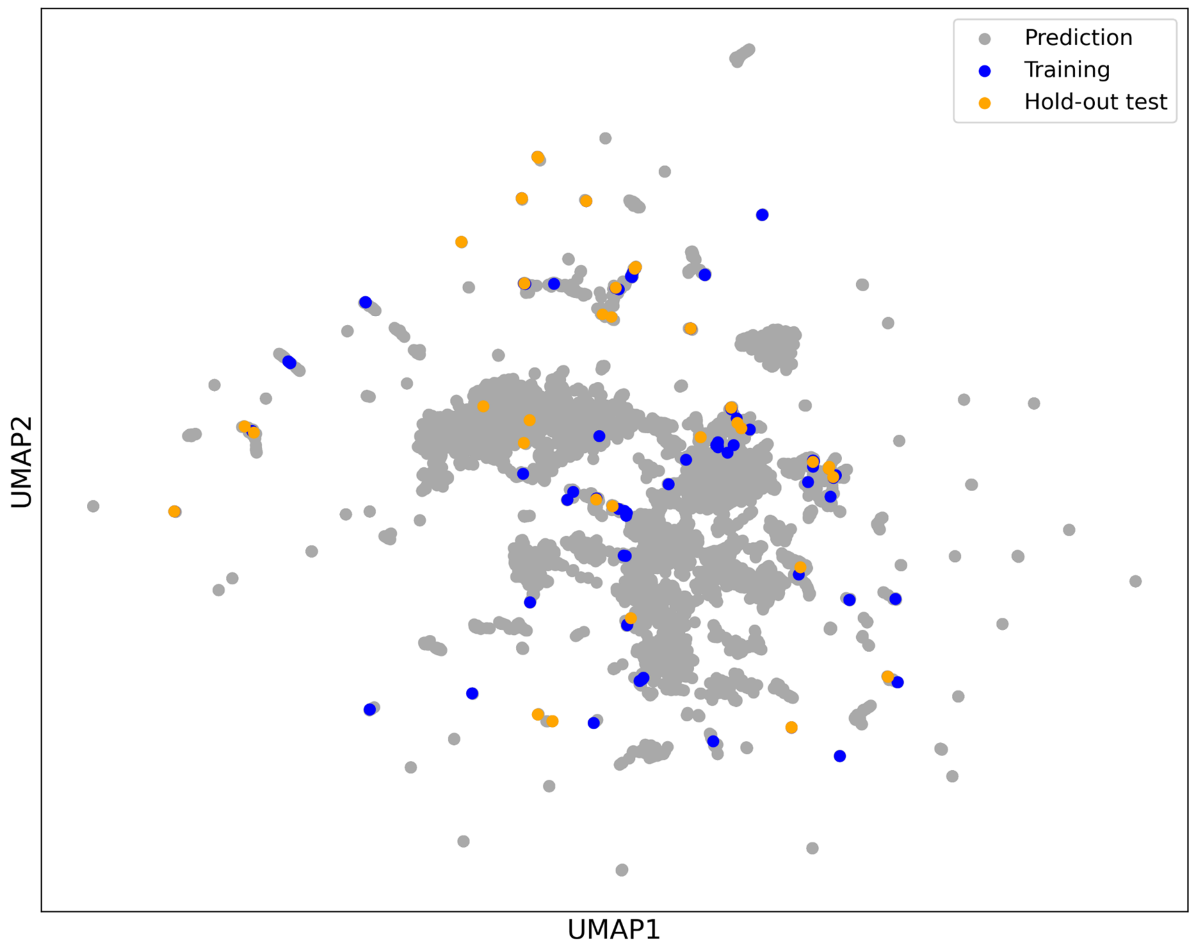

Figure S9. UMAP visualization of chemical space coverage for training and hold-out test PFAS. ECFP_6 fingerprints were used to map 140 PFAS structures within the broader chemical space of 10,553 PFAS structures (Prediction) of concern from U.S. EPA and NORMAN.

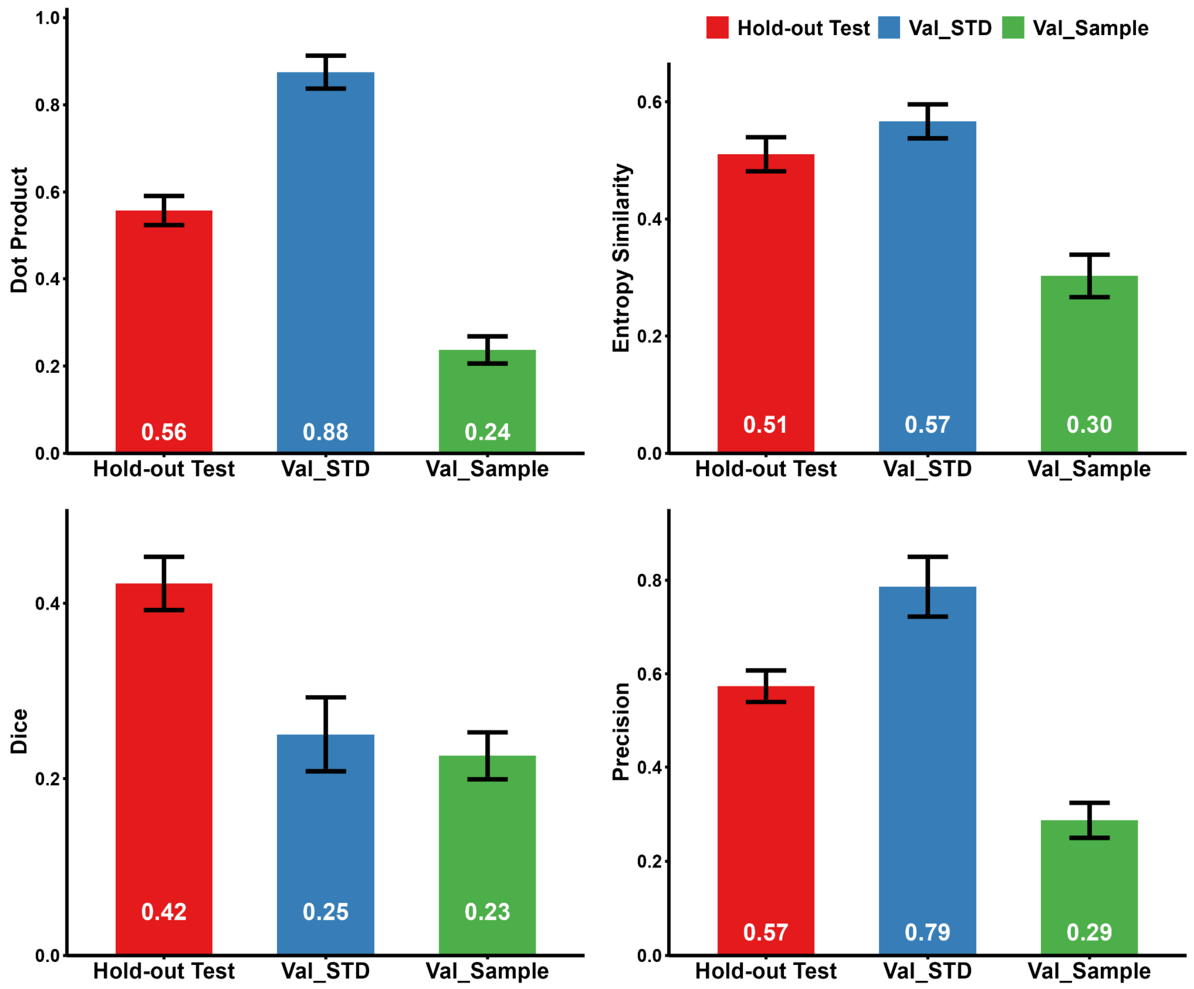

Figure S10. External validation of NPFAS-MS using two independent PFAS HRMS datasets. External validation was performed using an authentic standard-based dataset (Val_STD; 12 spectra)[4] and an environmental sample-based dataset (Val_Sample; 72 spectra).[5] All PFAS structures included in Val_STD and Val_Sample were excluded from the original training and hold-out test datasets. Bar plots show mean scores with standard error for four spectral similarity metrics: dot product, entropy similarity, dice coefficient, and precision. Val_STD showed comparable or better performance for metrics incorporating both *m/z* and intensity information, whereas Val_Sample showed lower performance across all four metrics. The lower performance observed for Val_Sample may reflect greater sample complexity, potential co-eluting compounds, uncertainty in the reference annotations, and the presence of structurally novel PFAS with fragmentation patterns that may be less well captured by NPFAS-MS.

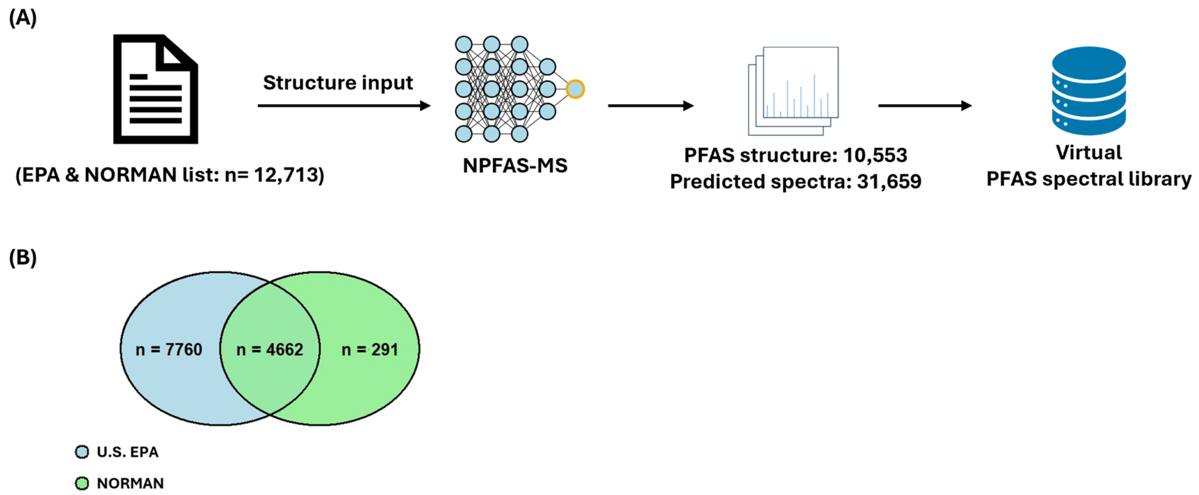

Figure S11. Construction of a virtual PFAS mass spectral library. (A) Workflow for virtual PFAS mass spectral library construction. PFAS structures from the U.S. EPA and NORMAN databases were processed through NPFAS-MS to generate 31,659 predicted MS/MS spectra, establishing a comprehensive virtual PFAS spectral library. (B) Illustrating the distribution and overlap of PFAS structures between the U.S. EPA CompTox Chemicals Dashboard (n=7,760 unique structures) and the NORMAN Substance Database (n=291 unique structures), with 4,662 structures common to both databases. The combined dataset yielded 12,713 unique PFAS structures after filtering and curation.

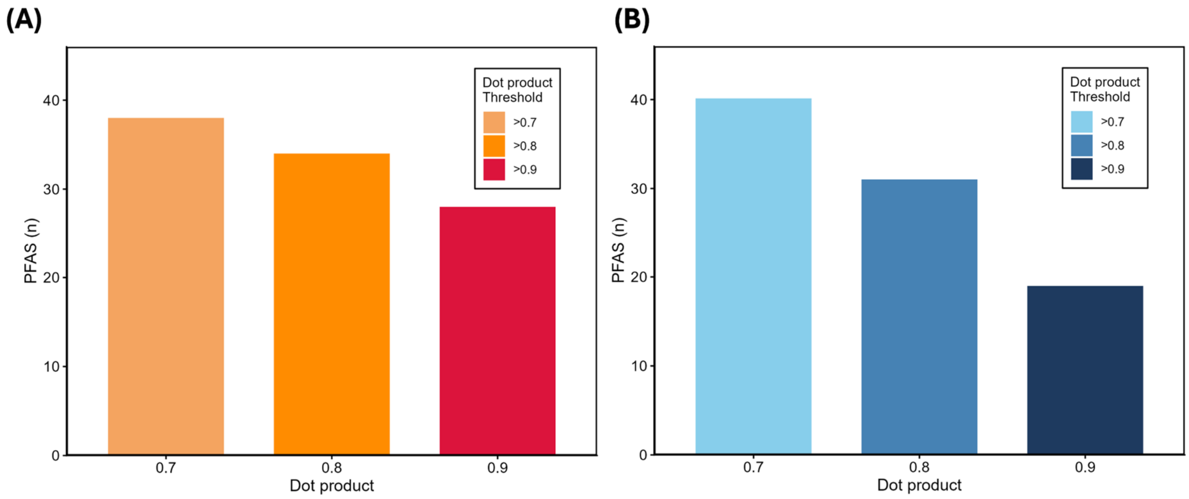

Figure S12. PFAS annotation performance at different dot product score thresholds in environmental samples. (A) Number of PFAS annotated in eight AFFF samples (AFFF-1 to AFFF-8) using the virtual library constructed with NPFAS-MS at different dot product score thresholds (> 0.7, > 0.8, > 0.9). (B) Number of PFAS annotated in pooled groundwater samples at different dot product score thresholds.

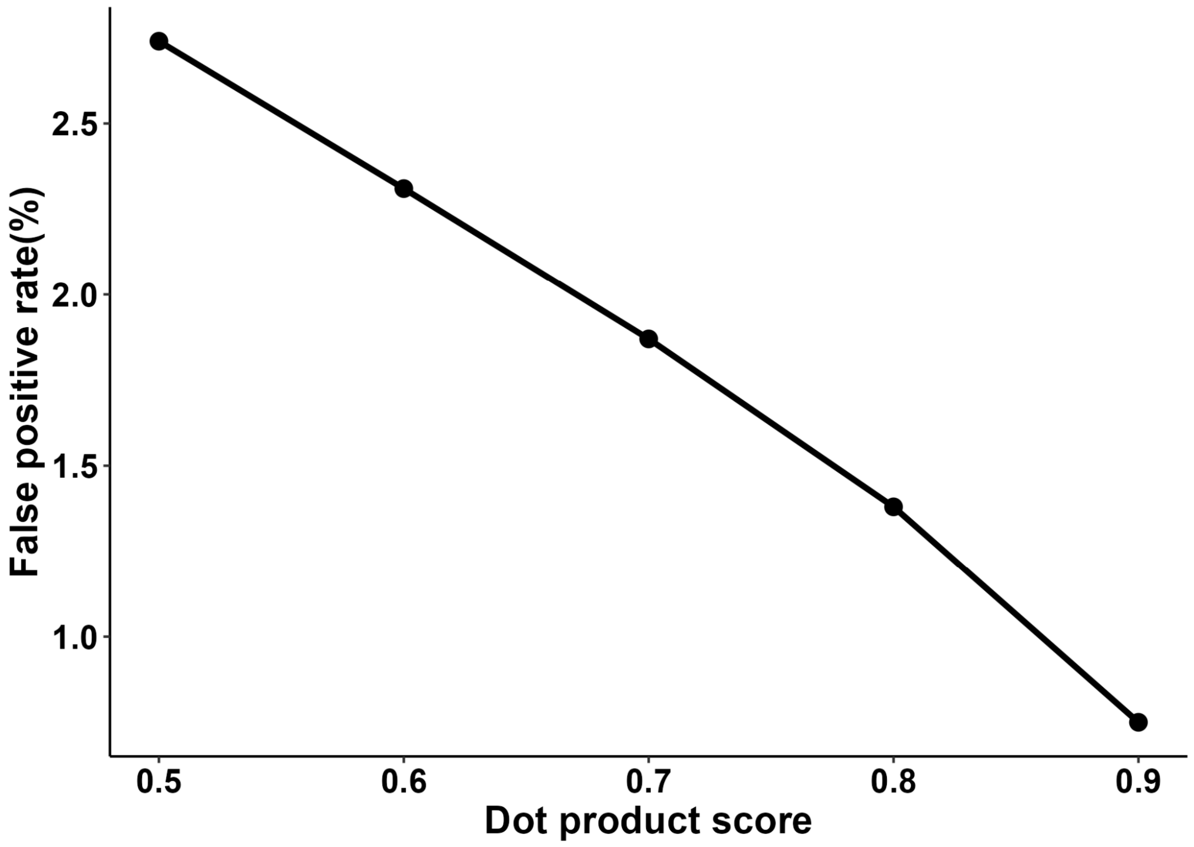

Figure S13. False positive rate of different dot product similarity thresholds using non-PFAS decoy dataset. The decoy dataset comprised 29,328 high-resolution MS/MS spectra from 5,840 non-PFAS compounds sourced from the MassBank of North America (MoNA), acquired in negative electrospray ionization (ESI) mode with $[M - H]^-$ precursor ions.

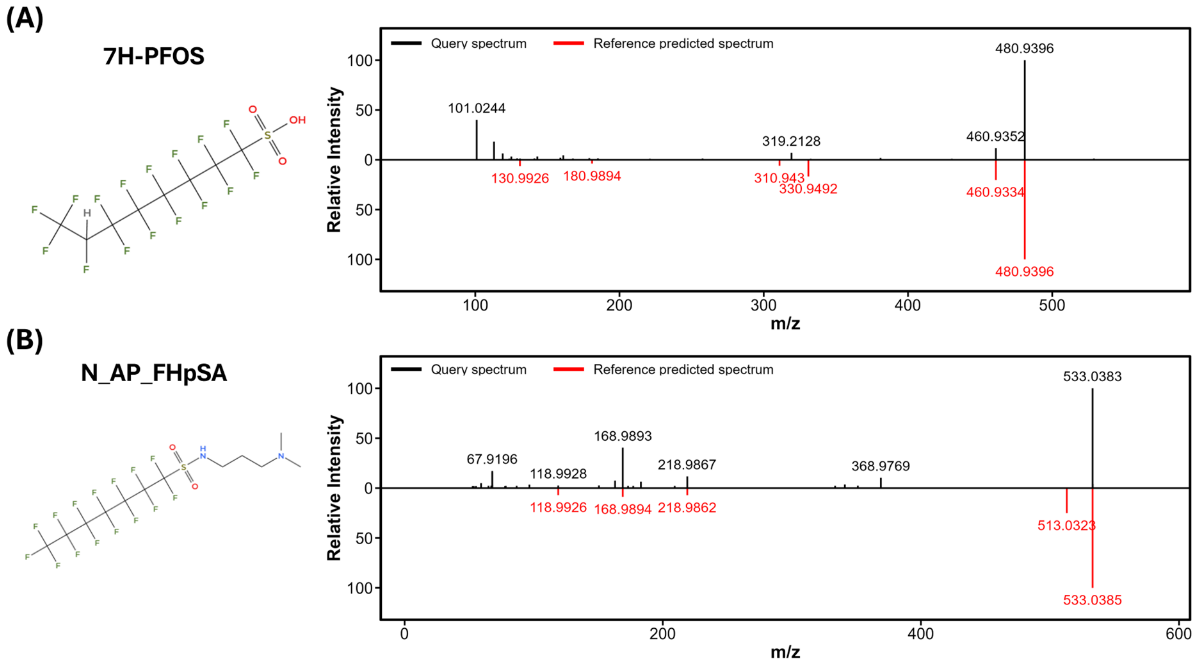

Figure S14. Byproduct PFAS detected in commercial AFFF products. (A) Query spectrum (black) and NPFAS-MS predicted spectrum (red) for 7H-perfluorooctanesulfonic acid (7H-PFOS), a hydrogen-substituted PFSA (dot-product score: 0.96). Molecular structure is shown on the left. (B) Spectra comparison for N-(3-(dimethylamino)propyl)perfluoroheptanesulfonamide (N_AP_FHpSA) (dot-product score: 0.89).

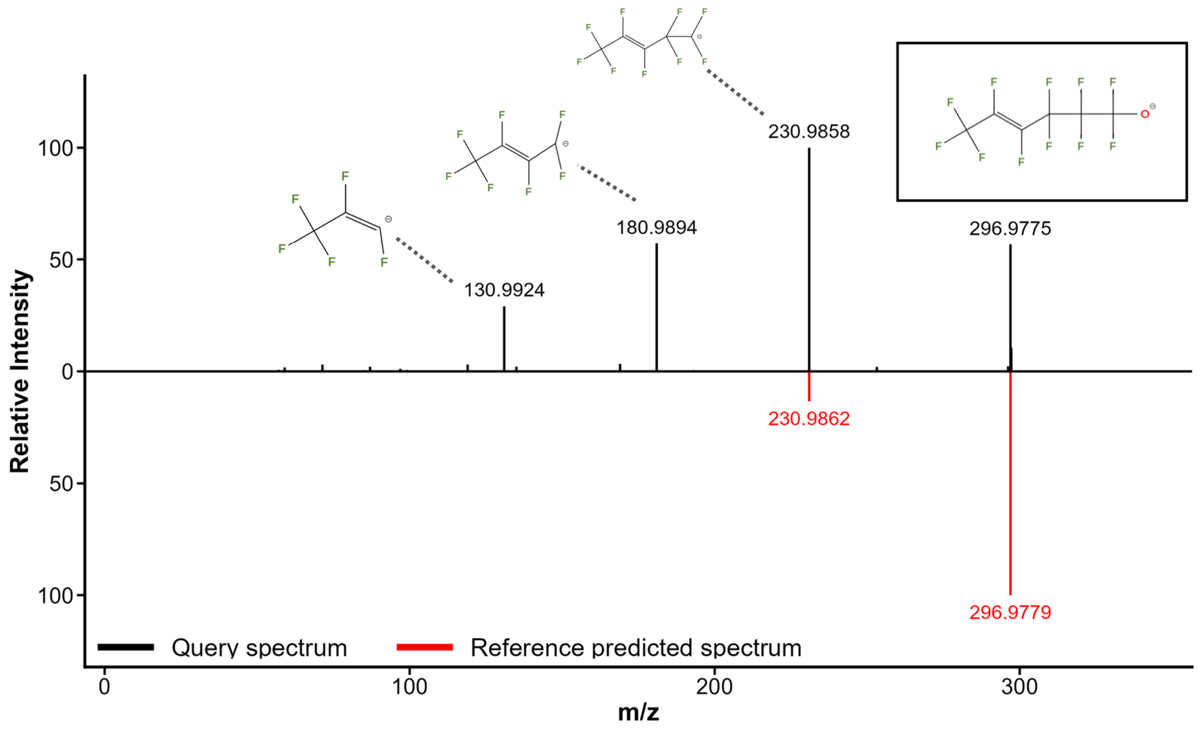

Figure S15. Structural annotation of (E)-5H-perfluorooct-6-ene-1-sulfonic acid (H-UPFOS) in AFFF products. Query spectrum from AFFF sample analysis (black) and NPFAS-MS predicted spectrum (red) for H-UPFOS (dot-product score: 0.92). The precursor ion ($[M - H]^-$, *m/z* 442.9427) and major product ions are labeled with proposed fragmentation structures. Successfully matched fragments (*m/z* 422.9369, 292.9826) were annotated using NPFAS-MS, while remaining product ions (*m/z* 79.9574) were structurally elucidated using MetFrag with NPFAS-MS-assigned molecular formulas.

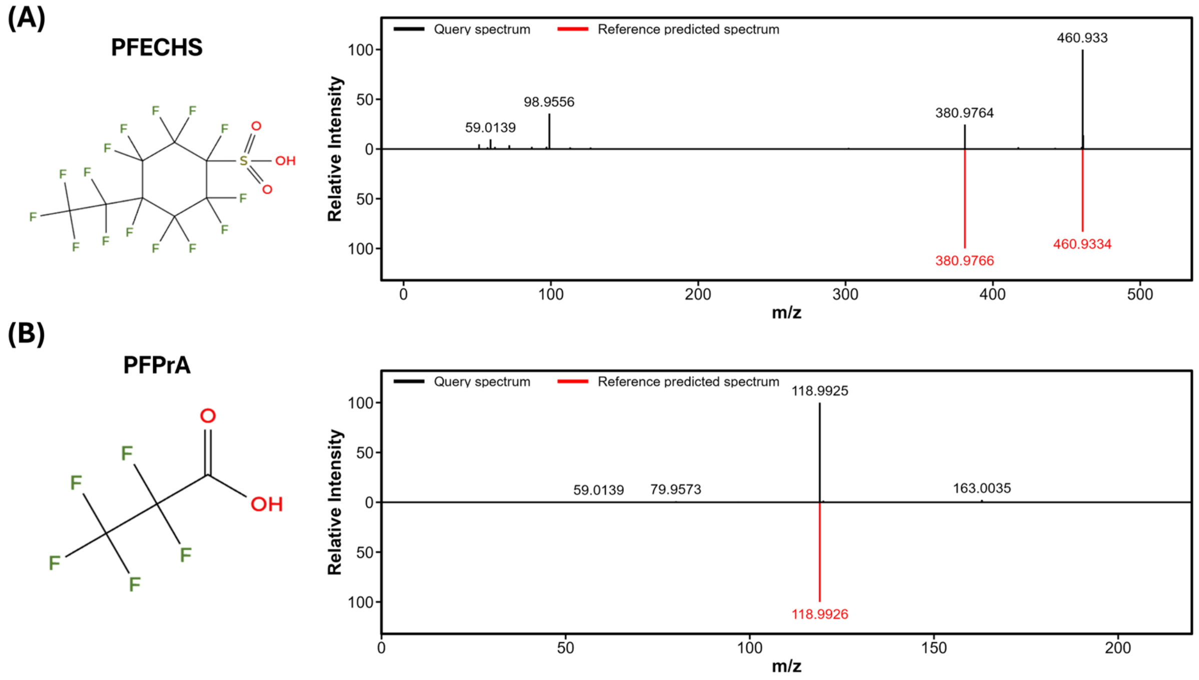

Figure S16. Representative emerging PFAS detected in groundwater samples. (A) Query spectrum (black) and NPFAS-MS predicted spectrum (red) for perfluoro-4-ethylcyclohexane sulfonic acid (PFECHS), a cyclic PFSA substitute for PFOS (dot-product score: 0.86). Molecular structure is shown on the left. (B) Spectra comparison for pentafluoropropanoic acid (PFPrA), an ultrashort-chain PFAS(dot-product score: 0.99).

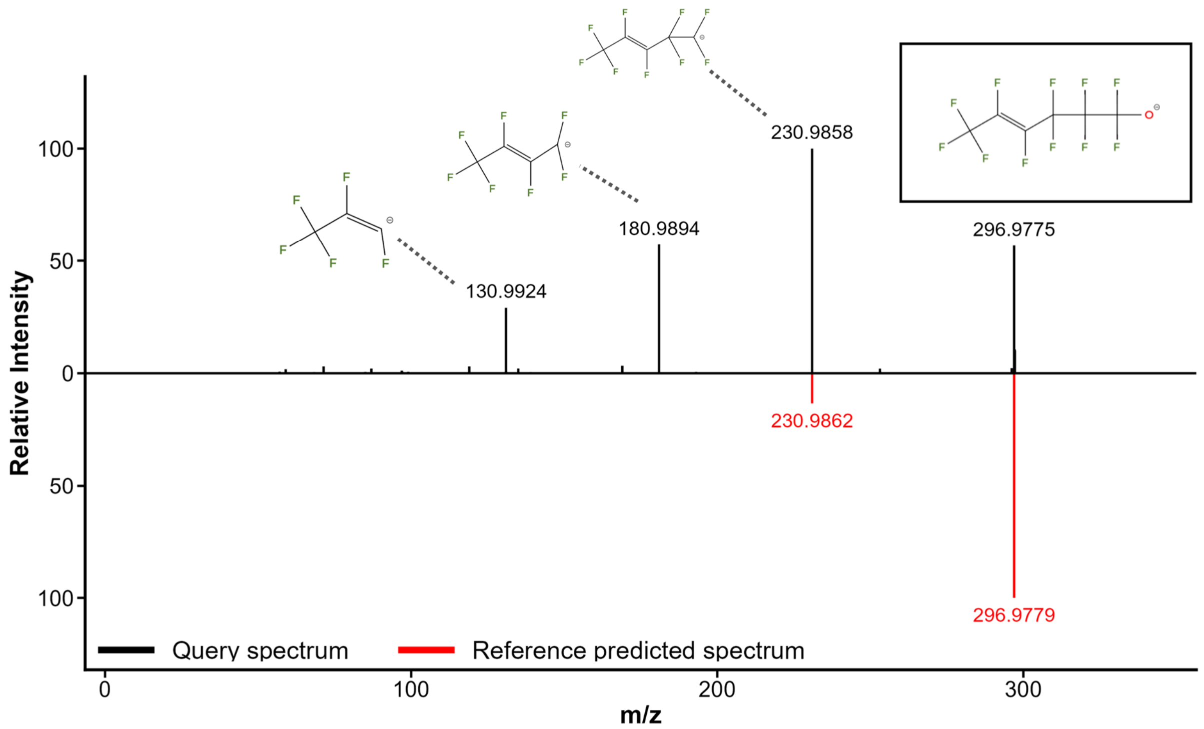

Figure S17. Structural annotation of (E)-1,1,2,2,3,3,4,5,6,6,6-undecafluorohex-4-en-1-ol (UPFHxA). Query spectrum from groundwater samples analysis (black) and NPFAS-MS predicted spectrum (red) for UPFHxA (dot-product score: 0.78). The precursor ion ($[M - H]^-$, *m/z* 296.9775) and major product ions are labeled. Successfully matched fragment (*m/z* 230.9858) was annotated using NPFAS-MS, while remaining product ions (*m/z* 130.9924, 180.9894) were structurally elucidated using MetFrag with NPFAS-MS-assigned molecular formula.

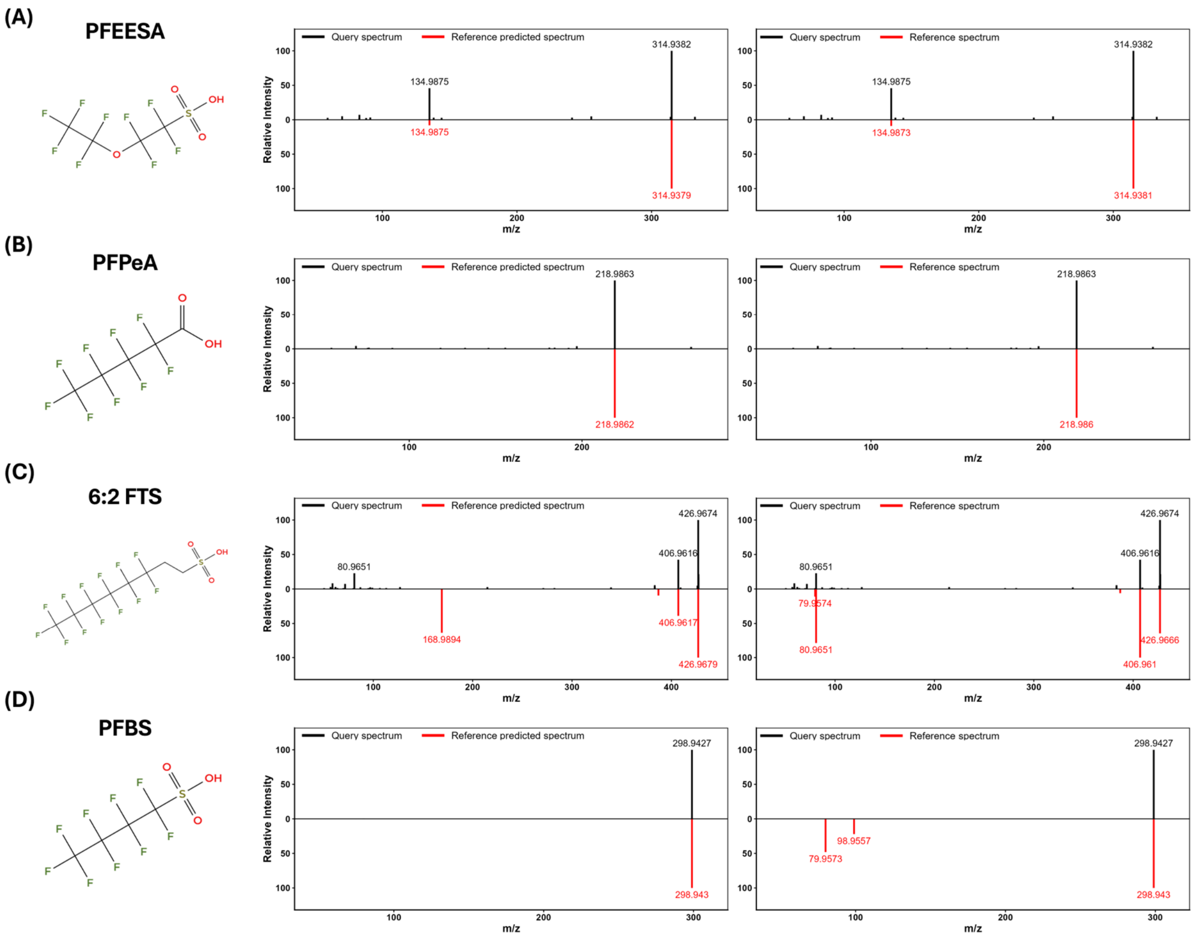

Figure S18. Validation of NPFAS-MS predicted spectra against reference standards across different environmental matrices. Representative PFAS annotated in environmental samples were validated using reference standards from the model training and testing sets. Left columns show comparisons between query spectra from environmental samples (black) and NPFAS-MS predicted spectra (red); right columns show comparisons between query spectra (black) and authentic reference standard spectra (red). (A) perfluoro(2-ethoxyethane)sulfonate (PFEESA) from the training set and (B) perfluoropentanoic acid (PFPeA) from the hold-out test set were detected in AFFF samples. (C) 6:2 fluorotelomer sulfonic acid (6:2 FTS) and (D) perfluorobutanesulfonic acid (PFBS), both from the training set, were detected in groundwater samples.

References

1. Frank, A. M.; Bandeira, N.; Shen, Z.; Tanner, S.; Briggs, S. P.; Smith, R. D.; Pevzner, P. A. Clustering millions of tandem mass spectra. *J. Proteome Res.* **2008,** *7* (1), 113-22, DOI: 10.1021/pr070361e
2. Wang, F.; Liigand, J.; Tian, S.; Arndt, D.; Greiner, R.; Wishart, D. S. CFM-ID 4.0: More Accurate ESI-MS/MS Spectral Prediction and Compound Identification. *Anal. Chem.* **2021,** *93* (34), 11692-11700, DOI: 10.1021/acs.analchem.1c01465
3. Wang, F.; Pasin, D.; Skinnider, M. A.; Liigand, J.; Kleis, J. N.; Brown, D.; Oler, E.; Sajed, T.; Gautam, V.; Harrison, S.; Greiner, R.; Foster, L. J.; Dalsgaard, P. W.; Wishart, D. S. Deep Learning-Enabled MS/MS Spectrum Prediction Facilitates Automated Identification Of Novel Psychoactive Substances. *Anal. Chem.* **2023,** *95* (50), 18326-18334, DOI: 10.1021/acs.analchem.3c02413
4. Wang, H.; Kuo, T.-C.; Tseng, Y. J. DeePFAS: Deep-Learning-Enabled Rapid Annotation of PFAS: Enhancing Nontargeted Screening through Spectral Encoding and Latent Space Analysis. *Environ. Sci. Technol.* **2025,** *59* (46), 24841-24852, DOI: 10.1021/acs.est.5c09769
5. Jiao, Z.; Taniyasu, S.; Yu, N.; Wang, X.; Yamashita, N.; Wei, S. Two-layer homolog network approach for PFAS nontarget screening and retrospective data mining. *Nat. Commun.* **2025,** *16* (1), 688, DOI: 10.1038/s41467-025-56035-1
